# An Optimized Machine Learning Model Accurately Predicts In-Hospital Outcomes at Admission to a Cardiac Unit

**DOI:** 10.3390/diagnostics12020241

**Published:** 2022-01-19

**Authors:** Sandeep Chandra Bollepalli, Ashish Kumar Sahani, Naved Aslam, Bishav Mohan, Kanchan Kulkarni, Abhishek Goyal, Bhupinder Singh, Gurbhej Singh, Ankit Mittal, Rohit Tandon, Shibba Takkar Chhabra, Gurpreet S. Wander, Antonis A. Armoundas

**Affiliations:** 1 Cardiovascular Research Center, Massachusetts General Hospital, Boston, MA 02129, USA; sbollepalli@mgh.harvard.edu (S.C.B.); kvkulkarni@mgh.harvard.edu (K.K.); 2 Department of Biomedical Engineering, Indian Institute of Technology Ropar, Rupnagar 140001, India; ashish.sahani@iitrpr.ac.in; 3 Department of Cardiology, Hero DMC Heart Institute, Unit of Dayanand Medical College and Hospital, Ludhiana 141001, India; drnavedaslam@yahoo.com (N.A.); bishav_68@yahoo.co.in (B.M.); drabhishekgoyal11@yahoo.in (A.G.); docbhupi@gmail.com (B.S.); singhgurbhej229@gmail.com (G.S.); drankitmittal@gmail.com (A.M.); drrohitt@yahoo.com (R.T.); shibbachhabra@yahoo.com (S.T.C.); drgswander@yahoo.com (G.S.W.); 4 Institute for Medical Engineering and Science, Massachusetts Institute of Technology, Cambridge, MA 02139, USA

**Keywords:** machine learning, mortality, duration of stay, heart failure, STEMI, pulmonary embolism

## Abstract

Risk stratification at the time of hospital admission is of paramount significance in triaging the patients and providing timely care. In the present study, we aim at predicting multiple clinical outcomes using the data recorded during admission to a cardiac care unit via an optimized machine learning method. This study involves a total of 11,498 patients admitted to a cardiac care unit over two years. Patient demographics, admission type (emergency or outpatient), patient history, lab tests, and comorbidities were used to predict various outcomes. We employed a fully connected neural network architecture and optimized the models for various subsets of input features. Using 10-fold cross-validation, our optimized machine learning model predicted mortality with a mean area under the receiver operating characteristic curve (AUC) of 0.967 (95% confidence interval (CI): 0.963–0.972), heart failure AUC of 0.838 (CI: 0.825–0.851), ST-segment elevation myocardial infarction AUC of 0.832 (CI: 0.821–0.842), pulmonary embolism AUC of 0.802 (CI: 0.764–0.84), and estimated the duration of stay (DOS) with a mean absolute error of 2.543 days (CI: 2.499–2.586) of data with a mean and median DOS of 6.35 and 5.0 days, respectively. Further, we objectively quantified the importance of each feature and its correlation with the clinical assessment of the corresponding outcome. The proposed method accurately predicts various cardiac outcomes and can be used as a clinical decision support system to provide timely care and optimize hospital resources.

## 1. Introduction

Patients with diverse cardiovascular diseases are admitted through the emergency department, into the wards, or to the cardiac care units depending on whether they are acutely sick or being admitted for further evaluation. In general, at each stage, patients are triaged by clinical professionals in order to provide timely care. At the same time, a large set of demographic and clinical parameters are being recorded for each patient, and manually analyzing and synthesizing information from all these variables proves to be challenging. In this context, it is imperative to develop a decision support system to assist clinicians in assessing patient risk, providing timely care, and optimizing resource utilization [1,2,3,4].

Various algorithms have been developed to predict in-hospital outcomes. These include mortality prediction systems, such as the acute physiology and chronic health evaluation (APACHE) score, the simplified acute physiology score (SAPS), and the sequential organ failure assessment (SOFA) score [5,6,7,8,9], the duration of stay estimation based on electronic health record data [10], and outcomes prediction-specific to underlying medical conditions [11,12]. However, these algorithms are tailored for subjects admitted to the intensive care units or general medical emergency departments and are not optimized to predict outcomes at the time of admission to the cardiac care unit. Further, these methods are developed using a small subset of all available parameters.

With an abundance of data being recorded, machine learning (ML) methods, which learn to discover patterns in large volumes of data, appear to be an attractive solution [13,14,15]. ML algorithms are known to process a large set of input parameters and remain flexible to predict various outcomes based on suitable training [16,17]. However, the major drawback with ML methods for large scale deployment in the medical domain is model interpretability [18,19].

In the present work, we used a machine learning model to predict in-hospital mortality, heart failure, ST-segment elevation myocardial infarction (STEMI), pulmonary embolism, and duration of stay using data available at the time of admission to a cardiac care unit. We optimized our algorithm to predict outcomes using all available parameters, including demographic and clinical parameters. Next, using permutation feature importance method [20], we objectively assigned importance scores for each feature to facilitate model interpretability. Favorably, most significant features for the ML performance in the present work are in agreement with the clinical understanding of the corresponding outcomes. In addition, using such objective importance scores, we excluded some of the redundant features, to further improve the model performance. Finally, we recursively excluded the most significant features and studied the objective importance scores assigned by the machine learning model to derive interesting clinical insights in case those features are not timely available.

In practice, our proposed method can aid in clinical decision to stratify risk, provide timely care, and improve resource utilization and hence the overall quality of care.

## 2. Methods

### 2.1. Dataset

The present study was conducted retrospectively on patients admitted over a period of two years (1 April 2017 to 31 March 2019) at Hero Dayanand Medical College Heart Institute Unit of Dayanand Medical College and Hospital, Ludhiana, Punjab, India. This is a tertiary care medical college and hospital. During the study period, the cardiology unit had 14,845 admissions corresponding to 12,258 patients. For 1921 patients who had multiple admissions, we considered the data from their last admission only. In addition, 760 patients who got discharged against medical advice were also excluded from the analysis. Records from the remaining 11,498 patients were used to obtain features and outcomes. We used the admission records to obtain parameters related to demographics, admission details, lab measurements, and comorbidities. The list of variables used in the present study along with the patients’ baseline characteristics of the study cohort are provided in the Table 1. We reported continuous features with mean (standard deviation) and median (interquartile range) values of the cohort, while categorical elements are reported as percentages. Information related to race was not collected, as all patients resided in India, and considered to be of the same race.

Specifically, data were related to patients′ date of admission; date of discharge; demographics, such as age, sex, locality (rural or urban); type of admission (emergency or outpatient); patient history, including smoking, alcohol, diabetes mellitus (DM), hypertension (HTN), prior coronary artery disease (CAD), prior cardiomyopathy (CMP), and chronic kidney disease (CKD); and lab parameters corresponding to hemoglobin (HB), total lymphocyte count (TLC), platelets, glucose, urea, creatinine, brain natriuretic peptide (BNP), raised cardiac enzymes (RCE) and ejection fraction (EF). Other comorbidities and features (28 features), including heart failure, STEMI, and pulmonary embolism, were recorded and analyzed. Among other comorbidities, shock was defined by systolic blood pressure <90 mmHg, and the cause for shock was due to any reason but cardiac. Patients in shock due to cardiac reasons were classified into cardiogenic shock, while patients in shock due to multifactorial pathophysiology (cardiac and non-cardiac) were considered for both categories. The outcomes indicating whether the patient is discharged or expired in the hospital were also recorded.

### 2.2. Outcomes

We are interested in predicting a wide range of outcomes, including in-hospital mortality, which is an important clinical outcome; the duration of hospital stay, which is a measure for resource utilization; and variable patient diagnoses, such as heart failure, STEMI, and pulmonary embolism. While STEMI and pulmonary embolism were newly occurring during hospitalization, heart failure could be newly occurring or an existing condition diagnosed during hospitalization. Specifically, we aim to predict the outcomes based on parameters acquired during admission and prior to the starting of treatment. We obtained the ground-truth annotation for mortality as a discharge disposition of expired. Duration of stay was obtained from the difference of the date of discharge and the date of admission. Heart failure, STEMI and pulmonary embolism were obtained from the clinical flag set in the diagnosis chart. We used all available features for predicting mortality and duration of stay. For classification of heart failure, STEMI, and pulmonary embolism, we only used patient demographics, admission type, patient history, and lab parameters while excluding comorbidities.

### 2.3. Performance Metrics

To estimate the performance of the proposed method, we performed k-fold cross-validation (with stratified random sampling) on the available data. We assessed the 10-fold cross-validation performance of our method and then took the mean performance along with the 95% confidence interval (CI) range. We considered only the data from the latest admission for each patient and ensured that each patient was included either in the training or in the test set. During each fold, only data from the fold-training set was used for tuning hyperparameter. In particular, a random 10% of the fold-training data was used as validation data to tune the hyperparameters, and the remaining 90% of the fold-training data was used for training the model. The resulting architecture with optimal hyperparameters was evaluated on the test set, and the mean performance across all folds was reported. We used AUC and mean absolute error to report performance of classification and regression models respectively. Further, we used the permutation-importance technique to obtain the importance score for each feature, indicating their contribution towards the model performance.

### 2.4. Data Preprocessing

All categorical variables were encoded as numerical. In particular, each binary variable was mapped to −1 and 1. Missing values in the data were imputed using the k-Nearest Neighbors (KNN) approach using Euclidean distance metric [21]. In particular, each missing feature was imputed using average feature value from k = 10 nearest neighbors. We normalized the data to have a zero mean and unit variance.

To perform the regression on the duration of stay data, we excluded values that exceeded the 15 days using the median based rejection method [22]; where the duration of stay values that are less than a factor 1.5 of the inter quartile range (IQR) below the 25th percentile (Q1 − 1.5 * IQR) or greater than a factor 1.5 of the IQR above the 75th percentile (Q3 + 1.5 * IQR), were excluded. Such data exclusion was performed only during model development, and the performance of the trained models was evaluated on the entire dataset. For imputation and normalization of the test and validation data sets in each fold, we used the parameters estimated from the training data of the corresponding fold.

### 2.5. Machine Learning Algorithm

We used a fully connected neural network algorithm for both classification and regression tasks [23]. In particular, the fully connected neural network architecture consists of multiple layers between input and output layers. Each layer has multiple nodes, and each node is connected to all the nodes in the next layer through a weight vector. These weights are learnt during network training using a backpropagation algorithm. For classification and regression tasks, we used binary crossentropy and mean absolute error as the cost functions, respectively. We developed our models using python (version 3.8.3) and the keras open-source library (version 2.4.0). We used the scikit-learn library for feature imputation and feature importance computation using KNNImputer and permutation_importance routines, respectively. Performance metrics were computed using MATLAB (version R2014b). Finally, we optimized the various hyper parameters of the network, as described below.

### 2.6. Network Optimization

We used the keras tuner library to optimize the architecture of the neural network [24]. Using a random grid search method [25], we chose the number of hidden layers between 1 and 10; the number of nodes in each layer were chosen within the range of 10 to 200 with a step size of 10. The activation function was chosen between sigmoid and ReLu, while the learning rate was chosen from 0.001 to 0.1, incremented by a factor of 10. We randomly sampled the hyper parameters over 100 trials while repeating each trial thrice. Finally, the optimization was performed on all 10-fold cross-validation data to obtain the optimal architecture. We obtained a different architecture for every fold, and we chose the architecture with minimum number of trainable parameters across 10 folds. We then re-trained the network using the training and validation sets of each fold and reported the mean performance on the test sets.

### 2.7. Performance Evaluation and Feature Selection

To evaluate the model performance, we first trained models that used all features (FS1) as inputs specific to each outcome. Next, we used a permutation-importance technique, and we obtained the importance score for each feature, indicating their contribution towards model performance. Based on the feature importance scores, we obtained a reduced feature set (FS2) by excluding those features with the cumulative importance contributing less than 1% to the overall importance. Excluding such redundant features is known to improve the model performance as well as reduce the computational complexity [26].

We carried out additional analysis on modified feature sets, where we omitted the most important features. This was motivated by the fact that the top features could be already established predictors of the relevant outcomes, and we were interested in determining how predictive the less obvious features were. Therefore, we excluded the most significant feature from FS2 to obtain feature set-3 (FS3); subsequently, excluding the most significant feature from FS3, we obtained feature set-4 (FS4). In the same vein, we obtained feature set-5 (FS5), feature set-6 (FS6), and feature set-7 (FS7) by recursively excluding the most significant feature from the corresponding super sets FS4, FS5, and FS6, respectively. Although such elimination of the most significant feature seems counterintuitive, due to potential decrease in model performance, the importance of the non-obvious features can be objectively quantified to derive further insights. Additionally, in practice, certain important features could be missing due to time and resource constrains, and excluding such features would also calibrate the model performance based on individual circumstances.

First, we obtained the baseline performance by optimizing the network configuration using FS1 as input. Next, we excluded the non-significantly contributing features from FS1 to obtain FS2 and again optimized the network configuration to obtain the performance with FS2 as input. We used the optimal configuration obtained for FS2 for training and evaluation of models developed using FS3–FS7. The optimal network configuration obtained for each outcome is described in the Supplementary Materials. The performance of the model on the feature sets FS1–FS7 is shown in Table 2. Best mean performance over 10-fold cross-validation was obtained for the models trained with FS2 (reduced/optimal feature set) as input for all the outcomes. A detailed description of the performance for each outcome is presented in the Supplementary Materials. Major conclusions specific to each outcome can be summarized as follows:

### 2.8. Mortality

**Figure 1:**
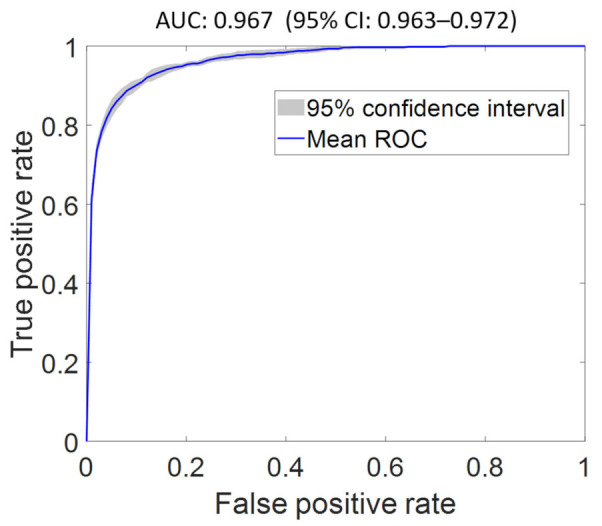
Optimal receiver operating characteristic curve of mortality classifier using the optimal feature set (FS2) as input. The proposed model achieved an AUC of 0.967 (95% CI: 0.963–0.927), which is superior to the AUC of the classifier using all features (FS1) as input.

We obtained a baseline AUC of 0.955 (95% CI: 0.947–0.963) using FS1 as input. An optimal AUC of 0.967 (95% CI: 0.963–0.972) was achieved using FS2 as input (see Figure 1). The optimized network architecture has one hidden layer with 150 nodes, sigmoid activation, and a learning rate of 0.01, with the top three features being EF, shock, and admission type. Indeed, EF and shock have been reported to predict mortality [27,28]. The feature importance score and receiver operator characteristic (ROC) curves for the classifier evaluated using FS1–FS7 are shown in the Supplementary Materials Figure S1A–G and in the Supplementary Materials Figure S6, respectively. The features of highest importance in predicting mortality using FS2–FS7 are EF, shock, cardiogenic shock, prior CAD, urea, and creatinine, respectively. Although admission type is consistently listed in the top three features, a clinical variable took precedence as the most important feature for various input combinations.

### 2.9. Heart Failure

**Figure 2:**
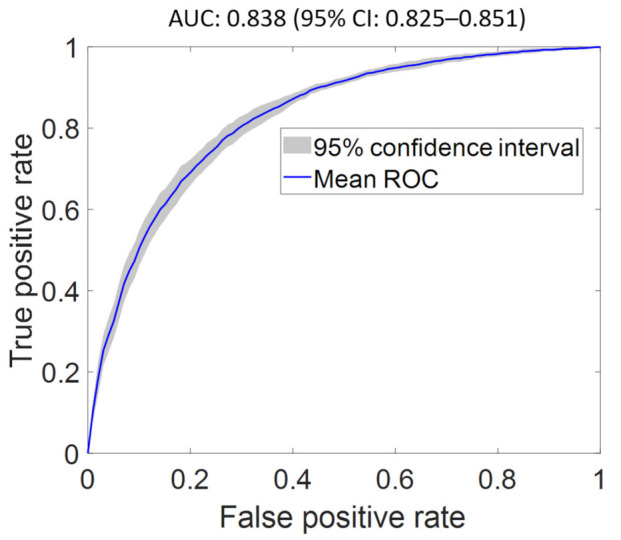
Optimal receiver operating characteristic curve of heart failure classifier using the optimal feature set (FS2) as input. The proposed model achieved an AUC of 0.838 (95% CI: 0.825–0.851), which is superior to the AUC of the classifier using all features (FS1) as input.

We obtained a baseline AUC of 0.833 (95% CI: 0.819–0.846) using FS1 as input. An optimal AUC of 0.838 (95% CI 0.825–0.852) was achieved using FS2 as input (see Figure 2). The optimized network architecture has one hidden layer with 140 nodes, sigmoid activation, and a learning rate of 0.01, with the top three features being BNP, EF, and urea. BNP and EF were the most significant features in detecting heart failure, correlating well with clinical knowledge [29]. When BNP and EF were excluded from model development (using FS5), prior CMP exhibited the highest importance. The feature importance score and ROC curves for the classifier evaluated using FS1–FS7 are shown in the Supplementary Materials Figure S2A–G and in the Supplementary Materials Figure S7, respectively. The features of highest importance in predicting heart failure using FS2–FS7 are BNP, EF, prior CMP, urea, creatinine, and admission type, respectively.

### 2.10. ST-Segment Elevation Myocardial Infraction

**Figure 3:**
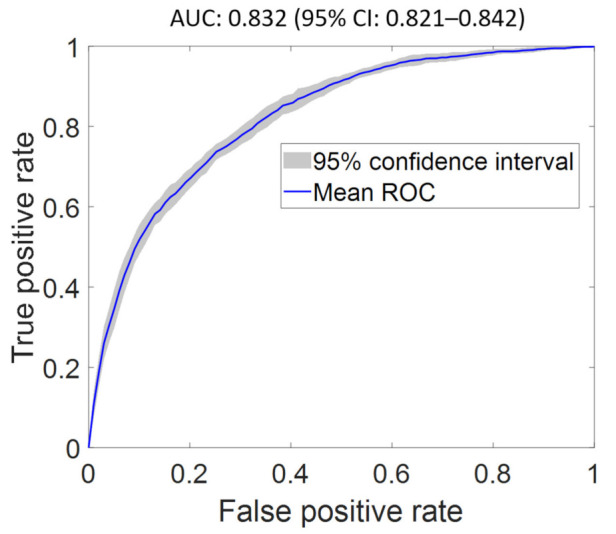
Optimal receiver operating characteristic curve of ST-segment elevation myocardial infarction (STEMI) classifier using the optimal feature set (FS2) as input. The proposed model achieved an AUC of 0.832 (95% CI: 0.821–0.842), which is comparable to the AUC of the classifier using all features (FS1) as input.

We obtained a baseline AUC of 0.832 (95% CI: 0.824–0.839) using FS1 as input. An optimal AUC of 0.832 (95% CI: 0.821–0.842) was achieved using FS2 as input (see Figure 3). The optimized network architecture has two hidden layers, each with dimension of 20 nodes, a ReLu activation, and a learning rate of 0.01, with the top three features being EF, prior CAD, and admission type. Indeed, STEMI and EF were significantly correlated [30], which is in agreement with reported data suggesting that reduced EF occurs in 30–40% of patients who suffer STEMI [31]. The feature importance score and ROC curves for the classifier evaluated using FS1–FS7 are shown in the Supplementary Materials Figure S3A–G and in the Supplementary Materials Figure S8, respectively. The features of highest importance in predicting STEMI objectively using FS2–FS7 are EF, prior CAD, admission type, total lymphocyte count (TLC), glucose, and age, respectively.

### 2.11. Pulmonary Embolism

**Figure 4:**
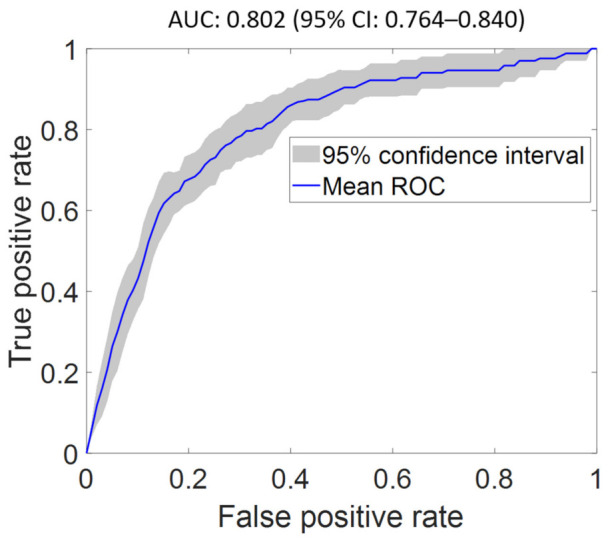
Optimal receiver operating characteristic curve of pulmonary embolism classifier using the optimal feature set (FS2) as input. The proposed model achieved an AUC of 0.802 (95% CI: 0.764–0.840), which is superior to the AUC of the classifier using all features (FS1) as input.

We obtained a baseline AUC of 0.779 (95% CI: 0.733–0.826) using FS1 as input. An optimal AUC of 0.802 (95% CI: 0.764–0.84) was achieved using FS2 as input (see Figure 4). The optimized network architecture has two hidden layers with dimension of 50 nodes and 80 nodes for layer 1 and layer 2, respectively, with sigmoid activation for both layers and a learning rate of 0.01, with the top three features being EF, prior CAD, and admission type. Indeed, pulmonary embolism and acute heart failure are known to be present concomitantly [32], which agrees with the clinical observations suggesting that the relative risk of pulmonary embolism is at least double to that of patients without heart failure and increases as LV systolic function declines [33], hence correlating well with EF. The feature importance score and ROC curves for the classifier evaluated using FS1–FS7 are shown in the Supplementary Materials Figure S4A–G and in the Supplementary Materials Figure S9, respectively. Features of highest importance in predicting pulmonary embolism objectively using FS2–FS7 are EF, prior CAD, admission type, locality, DM, and HTN, respectively.

### 2.12. Duration of Stay

**Figure 5:**
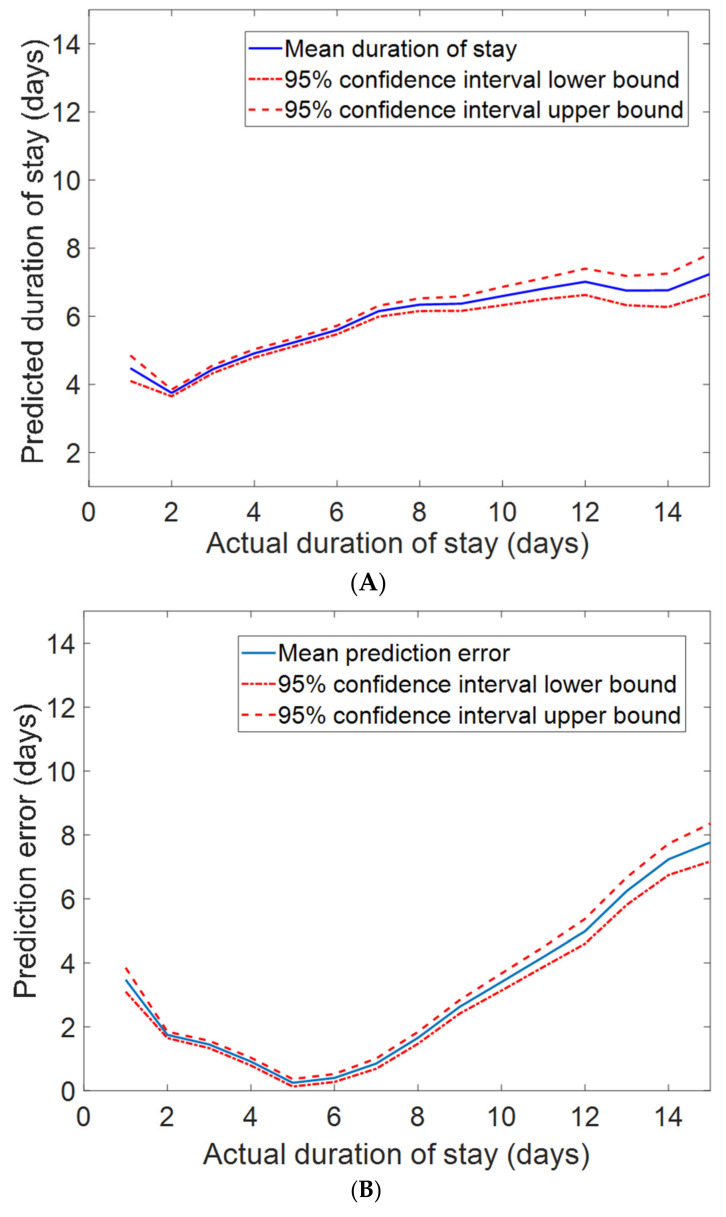
(**A**) The mean predicted duration of stay along with the 95% confidence intervals versus the actual duration of stay. (**B**) The absolute value of the mean prediction error along with the 95% confidence intervals versus the actual duration of stay. The proposed model achieved a mean absolute error (MAE) of 2.543 days (95% CI: 2.499–2.586), which is superior to the MAE of the classifier using all features (FS1) as input.

We obtained a baseline mean absolute error (MAE) of 2.561 (95% CI: 2.526–2.596) of data with a mean and median DOS of 6.35 days and 5.0 days, respectively, using FS1 as input. An optimal MAE of 2.543 (95% CI 2.499–2.586) was achieved using FS2 as input. The optimized network architecture has one hidden layer consisting of 10 nodes with ReLu activation and a learning rate of 0.01, with the top three features being admission type, TLC, and EF. An electronic health-record-based duration of stay estimation method reported a mean absolute error of 4.68 days [10] with a mean and median DOS of seven days and four days, respectively. The mean predicted DOS versus the actual DOS and the absolute value of the mean prediction error versus the actual DOS along with the corresponding 95% confidence intervals are shown in Figure 5A,B, respectively. Intuitively, admissions type has the highest importance, as emergency admissions may be related to a longer duration of stay. The feature importance score for models using FS1–FS7 as inputs are shown in the Supplementary Materials Figure S5A–G. Features of highest importance in estimating duration of stay objectively using FS2–FS7 are admission type, TLC, stable angina, EF, STEMI, and BNP, respectively.

## 3. Discussion

The present study demonstrates that a machine model can predict various clinical outcomes with high discriminatory performance. Although various scores exist for predicting the outcomes of critically ill patients in ICU, scores for stratifying risk at the admission in a cardiac unit emergency ward are limited. We proposed an optimized machine learning model to predict various outcomes based on available data during admission to a cardiac care unit. We also demonstrated that the features that contribute significantly in the machine learning algorithm performance are in agreement with the clinical knowledge of the underlying outcome. Several conclusions can be drawn from this study: *first*, a machine learning approach can predict various outcomes using the data available at the time of admission; *second*, the importance of various features in predicting the arrhythmia can be objectively quantified; *third*, such feature-importance scores can be used to explain machine learning models and hence corroborate with the clinical knowledge to build trust and facilitate practical deployment; and *fourth*, objective importance scores can provide interesting clinical insights in diagnosing various conditions.

Various methods have been reported to predict specific outcomes considered in the present study. The rapid emergency medicine score (REMS) was reported to predict in-hospital mortality in patients attending the emergency department with an AUC of 0.852 [34]. A method to predict mortality in departments of internal medicine reported an AUC of 0.857 [35]. A recent algorithm reported an AUC of 0.942 for predicting mortality at admission to a medical ward [36]. The present method achieved superior performance (AUC 0.967) compared to the reported methods in predicting mortality. Similarly, machine learning methods are being used in predicting heart failure [37], pulmonary embolism [38], mortality due to STEMI [39], and duration of hospital stay using electronic health record data [40]. However, these methods are not directly comparable, as we aim to predict the outcomes using only data available at the time of index admission to a cardiac care unit.

In the present work, we used different set of features as input to evaluate the performance of the classifier in various scenarios. In particular, we used all features (FS1) as input to obtain the baseline performance. Then, a reduced/optimal set of features (FS2) that provide the optimal performance was obtained and used thereafter. Finally, the most significant features from the optimal set were sequentially excluded (FS3–FS7) to assess the model performance when certain important features are missing due to practical constraints. Comparing FS1 and FS2 as inputs, the mean performance for FS2 is superior to FS1; however, the performance of 95% confidence interval (CI) values significantly overlapped for all outcomes except mortality. Such an observation is consistent with the reported studies that indicate the gain in performance using a reduced feature set is specific to the underlying outcome [26]. Using FS3–FS7 as input, as expected, resulted in performance decrease, as we sequentially excluded the most significant features. We observed that objective feature importance scores of the proposed machine learning models correlated well with clinical knowledge, establishing the confidence in the learnt models.

In general, admissions to these units are for patients at varied risk levels. Triaging the patients requiring quick decision making, that is based on the preponderance of patients’ clinical, historical, and lab tests is challenging, especially for the clinical staff at the admission unit. In this context, the proposed machine learning model that operates on data available at admission and is flexible to process varying feature inputs proves to be useful in providing timely care and optimizing the resources. Further, the features of importance in our models correlate well with the clinical state-of-art knowledge of the corresponding outcomes. In practice, the proposed system, when integrated into an admission ward, could serve as a decision support system to help triage patients and manage the available resources effectively.

## 4. Conclusions

In this study, we proposed a method to predict various outcomes based on data available at the time of admission to a cardiac care unit. In particular, we sought to accurately predict duration of stay, mortality, occurrence of heart failure, STEMI, and pulmonary embolism to facilitate patient risk assessment and to help triaging and optimizing resource utilization. To this end, we used a fully connected neural network algorithm to learn an optimal non-linear mapping of input features to the output. Using a permutation feature importance technique, we ranked the importance of each feature towards model performance. Next, we excluded some of the redundant features to further optimize the model performance. Using 10-fold cross-validation, our optimized machine learning model predicted mortality with a mean AUC of 0.967 (CI: 0.963–0.972), heart failure AUC of 0.838 (CI: 0.825–0.851), ST-segment elevation myocardial infarction AUC of 0.832 (CI: 0.821–0.842), pulmonary embolism AUC of 0.802 (CI: 0.764–0.84), and estimated the duration of stay with a mean absolute error of 2.543 days (CI: 2.499–2.586). Favorably, features important for the model performance correlated well with the clinical knowledge of the underlying outcome. Finally, using various subsets of features, we derived insights onto which parameters contributed most to specific outcomes. With suitable translation, our method can serve as a decision support system to triage the patients at the admission unit and optimize the resource allocation.

## 5. Study Limitations

In this study, the models we developed used only two years of data from a single center; therefore, the generalizability of the models across multiple centers and multiple years has to be investigated. Additionally, the study was conducted retrospectively, with the intent to prospectively integrate and evaluate the proposed method in a cardiac care unit. However, our demonstration on independent 10-fold cross-validation indicates that similarly built models could translate well to multi center settings as well as prospective evaluation. Thus, the overall benefit of triaging and resource optimization using the proposed method has to be suitably quantified and evaluated.

Finally, an inherent limitation of the current approach in predicting clinical outcomes using only data available at the time of admission is that the system (patient) is affected (by numerous interventions) following admission. Such interventions should be considered in future model implementations (using tools like recurrent neural networks), which allow one to make reliable long-term predictions.

## Figures and Tables

**Table 1:** Baseline patient characteristics.

Total Subjects: 11,498	Mean (Standard Deviation) or Proportion (%)	Median Value (Interquartile Range)	Missing Values (%)
*Demographics*			
Age (year)	60.81 (13.47)	62.00 (17)	0.00
Gender (male %)	63.58		0.00
Locality (urban %)	75.84		0.00
Admission type (emergency %)	67.81		0.00
Duration of stay (days)	6.35 (4.56)	5.00 (5)	0.00
Mortality (expiry %)	9.40		0.00
*History*			
Smoking	5.06		0.00
Alcohol	6.77		0.00
Diabetes mellitus	30.99		0.00
Hypertension	47.70		0.00
Prior coronary artery disease	66.69		0.00
Prior cardiomyopathy	14.33		0.00
Chronic kidney disease	8.66		0.00
*Lab parameters*			
Hemoglobin (g/dL)	12.32 (2.31)	12.50 (3.1)	1.81
Total lymphocyte count (K/uL)	11.41 (7.08)	10.00 (5.3)	1.98
Platelets (K/uL)	238.38 (103.11)	226.00 (116)	2.04
Glucose (mmol:L)	160.47 (82.67)	134.00 (88)	5.28
Urea (mg/dL)	47.82 (40.57)	34.00 (29)	1.69
Creatinine (mg/dL)	1.30 (1.16)	0.93 (0.6)	1.76
Brain natriuretic peptide (pg/mL)	785.96 (988.89)	432.00 (934)	59.91
Raised cardiac enzymes	20.26		0.00
Ejection fraction	44.13 (13.42)	44.00 (28)	10.51
*Comorbidities*			
Severe anemia	1.79		0.00
Anemia	16.69		0.00
Stable angina	9.08		0.00
Acute coronary syndrome	37.16		0.00
ST-segment elevation myocardial infarction	14.62		0.00
Atypical chest pain	3.07		0.00
Heart failure (HF)	26.75		0.00
HF with reduced ejection fraction	14.19		0.00
HF with normal ejection fraction	12.63		0.00
Valvular	3.41		0.00
Complete heart block	2.61		0.00
Sick sinus syndrome	0.70		0.00
Acute kidney injury	20.51		0.00
Cerebrovascular accident infract	2.83		0.00
Cerebrovascular accident bleed	0.42		0.00
Atrial fibrillation	4.87		0.00
Ventricular tachycardia	3.13		0.00
Paroxysmal supraventricular tachycardia	0.74		0.00
Congenital	1.13		0.00
Urinary tract infection	5.87		0.00
Neuro cardiogenic syncope	0.97		0.00
Orthostatic	0.82		0.00
Infective endocarditis	0.16		0.00
Deep-vein thrombosis	1.37		0.00
Cardiogenic shock	6.78		0.00
Shock	5.64		0.00
Pulmonary embolism	1.46		0.00
Chest infection	2.33		0.00

**Table 2:** Performance of the proposed method in terms of area under receiver operating characteristic curve (AUC) for predicting mortality, heart failure, ST-segment elevation myocardial infarction (STEMI), and pulmonary embolism and in terms of mean absolute error (MAE) for estimating the duration of stay for various set of input features. FS1 constitutes all the features. Features with cumulative importance of less than 1% are excluded from FS1 to form FS2. The most significant feature from FS2 is removed to form FS3. Similarly, FS4, FS5, and FS6 are formed by excluding the most significant feature from the corresponding super sets FS3, FS4, FS5, and FS6, respectively. Optimal performance (highlighted in bold) is obtained on feature set-2 (FS2) by excluding redundant features.

**Feature Set**	**Mortality**	**Heart Failure**	**STEMI**	**Pulmonary Embolism**	**Duration of Stay**
	AUC (95% CI)				MAE (95% CI)
FS1	0.955 (0.947–0.963)	0.833 (0.819–0.846)	0.832 (0.824–0.839)	0.779 (0.733–0.826)	2.561 (2.526–2.596)
FS2	**0.967 (0.963–0.972)**	**0.838 (0.825–0.851)**	**0.832 (0.821–0.842)**	**0.802 (0.764–0.840)**	**2.543 (2.499–2.586)**
FS3	0.952 (0.946–0.958)	0.795 (0.783–0.807)	0.790 (0.778–0.801)	0.737 (0.688–0.786)	2.572 (2.528–2.616)
FS4	0.938 (0.929–0.947)	0.767 (0.755–0.779)	0.731 (0.714–0.748)	0.630 (0.580–0.680)	2.623 (2.579–2.667)
FS5	0.922 (0.912–0.933)	0.725 (0.715–0.734)	0.678 (0.666–0.691)	0.621 (0.585–0.658)	2.642 (2.598–2.685)
FS6	0.911 (0.901–0.922)	0.707 (0.696–0.718)	0.647 (0.632–0.662)	0.597 (0.557–0.636)	2.651 (2.608–2.695)
FS7	0.907 (0.899–0.915)	0.670 (0.657–0.684)	0.624 (0.615–0.633)	0.589 (0.543–0.636)	2.694 (2.650–2.737)

## Data Availability

The training dataset will be available to any investigator upon request.

## Supplementary Materials

The following supporting information can be downloaded at: https://www.mdpi.com/article/10.3390/diagnostics12020241/s1, Figure S1: Feature importance scores for predicting mortality using (A) FS1; (B) FS2; (C) FS3; (D) FS4; (E) FS5; (F) FS6; (G) FS7; Figure S2: Feature importance scores for predicting heart failure using (A) FS1; (B) FS2; (C) FS3; (D) FS4; (E) FS5; (F) FS6; (G) FS7; Figure S3: Feature importance scores for predicting of ST-segment elevation myocardial infarction using (A) FS1; (B) FS2; (C) FS3; (D) FS4; (E) FS5; (F) FS6; (G) FS7; Figure S4: Feature importance scores for predicting of pulmonary embolism using (A) FS1; (B) FS2; (C) FS3; (D) FS4; (E) FS5; (F) FS6; (G) FS7; Figure S5: Feature importance scores for estimating duration of stay using (A) FS1; (B) FS2; (C) FS3; (D) FS4; (E) FS5; (F) FS6; (G) FS7; Figure S6: Comparison of receiver operation characteristic (ROC) curves of mortality classifier using feature sets FS1–FS7 as inputs. The classifier model using FS2 as input has superior performance over the model using FS1 as input, and the performance gradually decreases with input being varied from FS3 to FS7; Figure S7: Comparison of ROC curves of heart failure classifier using feature sets FS1–FS7 as inputs. The classifier model using FS2 as input has slightly better performance over the model using FS1 as input, and the performance gradually decreases with input being varied from FS3 to FS7; Figure S8: Comparison of ROC curves of ST-segment elevation myocardial infarction (STEMI) classifier using feature sets FS1–FS7 as inputs. The classifier model using FS2 as input is comparable to the model using FS1 as input, and the performance gradually decreases with input being varied from FS3 to FS7; Figure S9: Comparison of ROC curves of pulmonary embolism classifier using feature sets FS1–FS7 as inputs. The classifier model using FS2 as input has superior performance over the model using FS1 as input, and the performance gradually decreases with input being varied from FS3 to FS7.

## References

[1] Kletečka-Pulker M., Völkl-Kernstock S., Fassl A., Klager E., Willschke H., Klomfar S., Wochele-Thoma T., Schaden E., Atanasov A. (2021). Telehealth in Times of COVID-19: Spotlight on Austria. Healthcare.

[2] Massaro A., Galiano A., Scarafile D., Vacca A., Frassanito A., Melaccio A., Solimando A., Ria R., Calamita G., Bonomo M. Telemedicine DSS-AI Multi Level Platform for Monoclonal Gammopathy Assistance. Proceedings of the 2020 IEEE International Symposium on Medical Measurements and Applications (MeMeA).

[3] Massaro A., Maritati V., Savino N., Galiano A. Neural Networks for Automated Smart Health Platforms oriented on Heart Predictive Diagnostic Big Data Systems. Proceedings of the 2018 AEIT International Annual Conference.

[4] Plati D.K., Tripoliti E.E., Bechlioulis A., Rammos A., Dimou I., Lakkas L., Watson C., McDonald K., Ledwidge M., Pharithi R. (2021). A Machine Learning Approach for Chronic Heart Failure Diagnosis. Diagnostics.

[5] Escobar G.J., Greene J.D., Scheirer P., Gardner M.N., Draper D., Kipnis P. (2008). Risk-Adjusting Hospital Inpatient Mortality Using Automated Inpatient, Outpatient, and Laboratory Databases. Med. Care.

[6] Le Gall J.R., Lemeshow S., Saulnier F. (1993). A new Simplified Acute Physiology Score (SAPS II) based on a European/North American multicenter study. JAMA.

[7] Moreno R.P., Metnitz P.G.H., Almeida E., Jordan B., Bauer P., Campos R.A., Iapichino G., Edbrooke D., Capuzzo M., Le Gall J.-R. (2005). SAPS 3—From evaluation of the patient to evaluation of the intensive care unit. Part 2: Development of a prognostic model for hospital mortality at ICU admission. Intensive Care Med..

[8] Vincent J.-L., Moreno R., Takala J., Willatts S., De Mendonça A., Bruining H., Reinhart C.K., Suter P.M., Thijs L.G. (1996). The SOFA (Sepsis-related Organ Failure Assessment) score to describe organ dysfunction/failure. Intensive Care Med..

[9] Zimmerman J.E., Kramer A., McNair D., Malila F.M. (2006). Acute Physiology and Chronic Health Evaluation (APACHE) IV: Hospital mortality assessment for today’s critically ill patients. Crit. Care Med..

[10] Baek H., Cho M., Kim S., Hwang H., Song M., Yoo S. (2018). Analysis of length of hospital stay using electronic health records: A statistical and data mining approach. PLoS ONE.

[11] Chicco D., Jurman G. (2020). Machine learning can predict survival of patients with heart failure from serum creatinine and ejection fraction alone. BMC Med. Inform. Decis. Mak..

[12] Hizoh I., Domokos D., Banhegyi G., Becker D., Merkely B., Ruzsa Z. (2020). Mortality prediction algorithms for patients undergoing primary percutaneous coronary intervention. J. Thorac. Dis..

[13] Shilo S., Rossman H., Segal E. (2020). Axes of a revolution: Challenges and promises of big data in healthcare. Nat. Med..

[14] Sevakula R.K., Au-Yeung W.M., Singh J.P., Heist E.K., Isselbacher E.M., Armoundas A.A. (2020). State-of-the-Art Machine Learning Techniques Aiming to Improve Patient Outcomes Pertaining to the Cardiovascular System. J. Am. Heart Assoc..

[15] Efimov I.R., Fu S.N., Laughner J.I. (2021). Cardiac Bioelectric Therapy: Mechanisms and Practical Implications.

[16] Au-Yeung W.-T.M., Sahani A.K., Isselbacher E.M., Armoundas A.A. (2019). Reduction of false alarms in the intensive care unit using an optimized machine learning based approach. NPJ Digit. Med..

[17] Au-Yeung W.-T.M., Sevakula R.K., Sahani A.K., Kassab M., Boyer R., Isselbacher E.M., Armoundas A. (2021). Real-time machine learning-based intensive care unit alarm classification without prior knowledge of the underlying rhythm. Eur. Hear. J. Digit. Health.

[18] Vellido A. (2020). The importance of interpretability and visualization in machine learning for applications in medicine and health care. Neural Comput. Appl..

[19] Bazoukis G.J.H., Loscalzo J., Antman E.M., Fuster V., Armoundas A.A. (2022). The Inclusion of Augmented Intelligence in Medicine: A Framework for Successful Implementation. Cell Rep. Med..

[20] Altmann A., Toloşi L., Sander O., Lengauer T. (2010). Permutation importance: A corrected feature importance measure. Bioinformatics.

[21] Troyanskaya O.G., Cantor M., Sherlock G., Brown P.O., Hastie T., Tibshirani R., Botstein D., Altman R.B. (2001). Missing value estimation methods for DNA microarrays. Bioinformatics.

[22] McGill R., Tukey J.W., Larsen W.A. (1978). Variations of box plots. Am. Stat..

[23] Yegnanarayana B. (2009). Artificial Neural Networks.

[24] O’Malley T.A.B. Elie and Long, James and Chollet, François and Jin, Haifeng and Invernizzi, Luca and others. Keras Tuner. 2019. https://github.com/keras-team/keras-tuner.

[25] Bergstra J., Bengio Y. (2012). Random search for hyper-parameter optimization. J. Mach. Learn. Res..

[26] Karabulut E.M., Özel S.A., Ibrikci T. (2012). A comparative study on the effect of feature selection on classification accuracy. Procedia Technol..

[27] Kay G.L., Sun G.-W., Aoki A., Prejean C.A. (1995). Influence of ejection fraction on hospital mortality, morbidity, and costs for CABG patients. Ann. Thorac. Surg..

[28] Al Jalbout N., Balhara K.S., Hamade B., Hsieh Y.-H., Kelen G.D., Bayram J.D. (2019). Shock index as a predictor of hospital admission and inpatient mortality in a US national database of emergency departments. Emerg. Med. J..

[29] Bozkurt B., Hershberger R.E., Butler J., Grady K.L., Heidenreich P.A., Isler M.L., Kirklin J.K., Weintraub W.S. (2021). 2021 ACC/AHA Key Data Elements and Definitions for Heart Failure: A Report of the American College of Cardiology/American Heart Association Task Force on Clinical Data Standards (Writing Committee to Develop Clinical Data Standards for Heart Failure). Circ. Cardiovasc. Qual. Outcomes.

[30] Kiron V., George P. (2019). Correlation of cumulative ST elevation with left ventricular ejection fraction and 30-day outcome in patients with ST elevation myocardial infarction. J. Postgrad. Med..

[31] Chen Z.-W., Yu Z.-Q., Yang H.-B., Chen Y.-H., Qian J.-Y., Shu X.-H., Ge J.-B. (2016). Rapid predictors for the occurrence of reduced left ventricular ejection fraction between LAD and non-LAD related ST-elevation myocardial infarction. BMC Cardiovasc. Disord..

[32] Arrigo M., Huber L.C. (2021). Pulmonary Embolism and Heart Failure: A Reappraisal. Card. Fail. Rev..

[33] Beemath A., Stein P.D., Skaf E., Al Sibae M.R., Alesh I. (2006). Risk of Venous Thromboembolism in Patients Hospitalized with Heart Failure. Am. J. Cardiol..

[34] Olsson T., Terent A., Lind L. (2004). Rapid Emergency Medicine score: A new prognostic tool for in-hospital mortality in nonsurgical emergency department patients. J. Intern. Med..

[35] Schwartz N., Sakhnini A., Bisharat N. (2017). Predictive modeling of inpatient mortality in departments of internal medicine. Intern. Emerg. Med..

[36] Soffer S., Klang E., Barash Y., Grossman E., Zimlichman E. (2021). Predicting In-Hospital Mortality at Admission to the Medical Ward: A Big-Data Machine Learning Model. Am. J. Med..

[37] Bazoukis G., Stavrakis S., Zhou J., Bollepalli S.C., Tse G., Zhang Q., Singh J.P., Armoundas A.A. (2021). Machine learning versus conventional clinical methods in guiding management of heart failure patients—A systematic review. Hear. Fail. Rev..

[38] Banerjee I., Sofela M., Yang J., Chen J.H., Shah N.H., Ball R., Mushlin A.I., Desai M., Bledsoe J., Amrhein T. (2019). Development and Performance of the Pulmonary Embolism Result Forecast Model (PERFORM) for Computed Tomography Clinical Decision Support. JAMA Netw. Open.

[39] Li X., Liu H., Yang J., Xie G., Xu M., Yang Y. (2017). Using Machine Learning Models to Predict In-Hospital Mortality for ST-Elevation Myocardial Infarction Patients. Stud. Health Technol. Inform..

[40] Carter E.M., Potts H.W.W. (2014). Predicting length of stay from an electronic patient record system: A primary total knee replacement example. BMC Med. Inform. Decis. Mak..

