# Supplementary material for: An Optimized Machine Learning Model Accurately Predicts In-Hospital Outcomes at Admission to a Cardiac Unit
Source: Diagnostics (Basel). 2022 Jan 19;12(2):241. doi: 10.3390/diagnostics12020241 (PMC8871182; doi:10.3390/diagnostics12020241)
Supplement: Supplementary file 1 [file diagnostics-12-00241-s001.zip › Supplement_Figures.pdf]

An Optimized Machine Learning Model Accurately Predicts in-Hospital Outcomes at Admission to a Cardiac Unit

Sandeep C. Bollepalli, PhD, Ashish Sahani, PhD, Naved Aslam, MD DM, Bishav Mohan, MD DM, Kanchan Kulkarni, PhD, Abhishek Goyal, MD DM, Bhupinder Singh, MD DM, Gurbhej Singh, MD DM, Ankit Mittal, MD DM, Rohit Tandon, MD, Shibba Takkar Chhabra, MD DM, Gurpreet S. Wander, MD, DM, Antonis A. Armoundas, PhD

Network optimization

As alluded in the manuscript, we used the keras tuner library to optimize the architecture of the neural network[1]. We optimized the architecture in two stages. First, using all the available variables as input to the network, we optimized the architecture. Next, based on the feature importance scores we excluded the features that contribute less than 1% to the classification performance. We considered a subset of features and optimized the network again. We repeated the optimization for 10 times using k-fold cross validation sets.

Model performance

Mortality

After optimizing the network using the keras tuner, using all available features as input (FS1), we obtained one hidden layer architectures with 40 nodes, followed by sigmoid activation with a learning rate of 0.1. Using this network, we obtained an AUC of 0.955 (95% CI: 0.947-0.963) for mortality prediction. The top three features of importance (in the decreasing order) are shock, EF and admission type. Using FS2, we again optimized the network architecture and obtained a

model with one hidden layer consisting of 150 nodes with sigmoid activation and a learning rate of 0.01. Using the optimized network, we achieved an AUC of 0.967 (95% CI: 0.963-0.972). Though, the top three features (EF, Shock and admission type) remained the same, EF now has higher importance over shock. Next, we sequentially excluded the top performing feature to understand which features contribute to the mortality prediction. The performance of the classifier by sequentially excluding the top performing features (FS3-FS7) are shown in Table -1. The feature importance values for all the configurations are shown in the Online Supplement Figures 1A- 1G. After excluding EF from FS2 to form FS3 as input, we achieved an AUC of 0.952 (95% CI: 0.946-0.958) and the mean importance of shock has almost doubled. Next, we excluded shock from FS3 to obtain FS4 as input and achieved an AUC of 0.938 (95% CI: 0.929-0.947). Interestingly, cardiogenic shock has highest importance now (previously ranked at 21 out of 24 features). Indeed cardiogenic shock and shock are clinically known to correlate well with the mortality. After excluding cardiogenic shock to form FS5 as input, we achieved an AUC of 0.922 (95% CI: 0.912-0.933), now prior CAD, urea, admission type, prior CMP and ACS assumes significance in predicting mortality with similar importance scores. Excluding prior CAD to form FS6, we achieved an AUC of 0.911 (95% CI: 0.901-0.922) with the highest importance assigned to urea. Excluding urea from FS6 resulted in FS7 with an AUC of 0.907 (95% CI: 0.899-0.915), now prior CMP has highest importance followed by creatinine (previously ranked at 7 out of 21 features), indicating the importance of urea and creatinine in their contribution towards mortality prediction.

In summary, EF and shock are the most significant features for predicting mortality objectively, which correlated well with established clinical knowledge[2,3]. With recursive feature elimination, we identified that cardiogenic shock, prior CAD, urea, prior CMP and creatinine values are the remaining features with most significance in predicting mortality.

Heart failure

After optimizing the network using keras tuner, using all available features as input (FS1), the optimal network configuration consists of one hidden layer of dimension 170, with sigmoid activation and learning rate of 0.01. Using such network, we obtained an AUC of 0.833 (95% CI:

0.819-0.846). BNP and EF features clearly standout in predicting HF incidence. Using FS2 as input, we again optimized the network architecture and obtained a model with one hidden layer consisting of 140 nodes, sigmoid activation and a learning rate of 0.01. Using the optimized network, we achieved an AUC of 0.838 (95% CI: 0.825-0.852). With the subset of features, not surprisingly BNP and EF are again the significant features in predicting HF. Next, we sequentially excluded the top performing feature to understand which features assume importance in classification. The feature importance values for all the input configurations are shown in the Online Supplement Figures 2A-2G. After excluding BNP from FS2 to form FS3 as input, we achieved an AUC of 0.795 (95% CI: 0.783-0.807) with the median importance of EF increased significantly from 0.16 to 0.67, and the next important feature after EF is urea. However, after excluding EF from FS3 to form FS4 as input, we obtained an AUC of 0.767 (95% CI: 0.755-0.779) with the highest importance assigned to prior CMP (previously ranked at 8 out of 10 features), followed by urea. Excluding prior CMP from FS4 to form FS5, urea now has the highest importance, with an AUC of 0.725 (95% CI: 0.715-0.734). In absence of urea, i.e., using FS6, creatinine has the highest significance in predicting HF with an AUC of 70.67%. Finally, we excluded creatinine and found that admission type has highest score in predicting HF with an AUC of 0.670 (95% CI: 0.657-0.684).

In summary, BNP and EF are key features of this model and established markers of HF [4]. However, in their absence, prior CMP, urea and creatinine are the remaining most significant features, in HF classification.

ST-segment elevation myocardial infarction

After optimizing the network using the keras tuner, using all available features as input (FS1), the optimal network configuration consists of one hidden layer of dimension 50, with sigmoid activation and learning rate of 0.01. Using such network, we obtained an AUC of 0.832 (95% CI: 0.824-0.839). EF has significant importance followed by prior CAD and admission type. Using FS2 as input, we again optimized the network architecture and obtained a model having two hidden layers, each with dimension of 20 nodes each with a relu activation and a learning rate of 0.01. Using the optimized network, we achieved an AUC of 0.832 (95% CI: 0.821-0.842,

with the top three features remaining the same. Next, we sequentially excluded the top performing feature to understand which features assume importance in classification. The feature importance values for all the input configurations are shown in the Online Supplement Figures 3A-3G. Excluding EF to form FS3 as input, we achieved an AUC of 0.790 (95% CI: 0.778-0.801) with prior CAD being the most important feature. Excluding prior CAD from FS3 to form FS4 as input, STEMI can be predicted with 0.731 (95% CI: 0.714-0.748) AUC with admission type having the highest significance followed by TLC and age. Next, subsequent elimination of top performing features resulted in FS5, FS6 and FS7, with high importance for TLC, glucose and age; and the corresponding AUCs are 0.678 (95% CI: 0.666-0.691), 0.647 (95% CI: 0.632-0.662) and 0.624 (95% CI: 0.615-0.633) respectively.

In summary, EF is the most significant feature of this model associated with STEMI [5]. In the absence of EF measurement, prior CAD, admission type, TLC, glucose and age are the remaining most significant features, in STEMI classification.

Pulmonary Embolism

After optimizing the network using the keras tuner, using all available features as input (FS1), the optimal network configuration consists of one hidden layer of dimension 80, with sigmoid activation and learning rate of 0.1. Using such network, we obtained an AUC of 0.779 (95% CI: 0.733-0.826). EF is the most significant feature, followed by prior CAD and TLC. Using FS2 as input, we again optimized the network architecture and obtained a model having two hidden layers with dimension of 50 nodes and 80 nodes respectively for layer 1 and layer 2, with sigmoid activation for both layers and a learning rate of 0.01. Using the optimized network, we achieved an AUC of 0.802 (95% CI: 0.764-0.84). With the top three features being EF, prior CAD and admission type. Next, we sequentially excluded the top performing feature to understand which features assume importance in classification. The feature importance values for all the input configurations are shown in the Online Supplement Figures 4A-4G. Excluding EF from FS2 to form FS3 as input, we achieved an AUC of 0.737 (95% CI: 0.688-0.786) with the median importance of prior CAD increased from 0.22 to 0.58. Excluding prior CAD from FS3 to form FS4 as input, we achieved an AUC of 0.630 (0.580-0.680) with admission type exhibiting the highest

significance followed closely by locality and HTN. Next, subsequent sequential elimination of top performing features resulted in FS5, FS6 and FS7 as inputs with high importance for locality, DM and HTN; and the corresponding AUCs are 0.621 (0.585-0.658), 0.597 (0.557-0.636) and 0.589 (0.543-0.636) respectively.

In summary, EF is the most significant feature of this model in predicting pulmonary embolism ,[6] being in agreement with clinical observations suggesting that the relative risk of pulmonary embolism is at least double to that of patients without heart failure, and increases as LV systolic function declines.[7] In the absence of EF measurement, prior CAD, admission type, locality, DM and HTN are the remaining most significant features, in pulmonary embolism classification.

Duration of hospital stay

After optimizing the network using the keras tuner, using all the available features as input (FS1), the optimal network configuration consists of one hidden layer of dimension 10, with relu activation and learning rate of 0.01. Using such network, we obtained the mean absolute error (MAE) in predicting the duration of stay as 2. 561 (95% CI: 2.526-2.596) days. The top three features of importance in predicting the duration of stay are stable angina, admission type and TLC. Using FS2 as input, we again optimized the network architecture and obtained a model with one hidden layer consisting of 10 nodes with relu activation and a learning rate of 0.01. Using the optimized network, we achieved an MAE of 2.543 (95% CI 2.499-2.586), with the top three features being admission type, TLC and EF. The feature importance values for all the input configurations are shown in the Online Supplement Figures 5A-5G. Intuitively, admission to an emergency department may lead to longer duration of stay and has higher importance. After excluding the admission type from FS2 to form FS3, we achieved a MAE of 2.572 (2.528-2.616) with TLC being the most importance features followed by stable angina. Excluding TLC from FS3 to form FS4 as input resulted in stable angina being the important feature for estimation of the duration of stay with MAE of 2.623 (2.579-2.667). Excluding stable angina from FS4 to form FS5, EF followed by STEMI assumes significance with MAE of 2.642 (2.598-2.685). Excluding EF

resulted in STEMI followed by BNP (previously ranked at 5 out of 26 features) as important factors for DOS estimation with MAE of 2.651 (2.608-2.695). Excluding STEMI, BNP has the highest importance in estimating DOS with MAE of 2.694 (2.650-2.737). Indeed, EF and BNP are highly correlated with heart failure and subjects with HF are bound to have a longer duration of stay.

In summary, admission type is the most significant feature in predicting the duration of hospital stay. With recursive feature elimination, TLC, stable angina, EF, STEMI and BNP are the remaining most significant features, in predicting the duration of stay.

References

1 O'Malley, T. a. B., Elie and Long, James and Chollet, François and Jin, Haifeng and Invernizzi, Luca and others. *Keras Tuner*, <https://github.com/keras-team/keras-tuner> (2019).

2 Kay, G. L., Sun, G. W., Aoki, A. & Prejean, C. A., Jr. Influence of ejection fraction on hospital mortality, morbidity, and costs for CABG patients. *Ann Thorac Surg* **60**, 1640-1650 (1995).

3 Al Jalbout, N. *et al.* Shock index as a predictor of hospital admission and inpatient mortality in a US national database of emergency departments. *Emergency Medicine Journal* **36**, 293-297, doi:10.1136/emermed-2018-208002 (2019).

4 Bozkurt, B. *et al.* 2021 ACC/AHA Key Data Elements and Definitions for Heart Failure: A Report of the American College of Cardiology/American Heart Association Task Force on Clinical Data Standards (Writing Committee to Develop Clinical Data Standards for Heart Failure). *Circulation: Cardiovascular Quality and Outcomes* **14**, e000102, doi:doi:10.1161/HCQ.0000000000000102 (2021).

5 Kiron, V. & George, P. V. Correlation of cumulative ST elevation with left ventricular ejection fraction and 30-day outcome in patients with ST elevation myocardial infarction. *J Postgrad Med* **65**, 146-151, doi:10.4103/jpgm.JPGM_364_18 (2019).

6 Arrigo, M. & Huber, L. C. Pulmonary Embolism and Heart Failure: A Reappraisal. *Card Fail Rev* **7**, e03-e03, doi:10.15420/cfr.2020.26 (2021).

7 Beemath, A., Stein, P. D., Skaf, E., Al Sibae, M. R. & Alesh, I. Risk of venous thromboembolism in patients hospitalized with heart failure. *Am J Cardiol* **98**, 793-795 (2006).

Online Supplement Figure Captions

Figure S1A

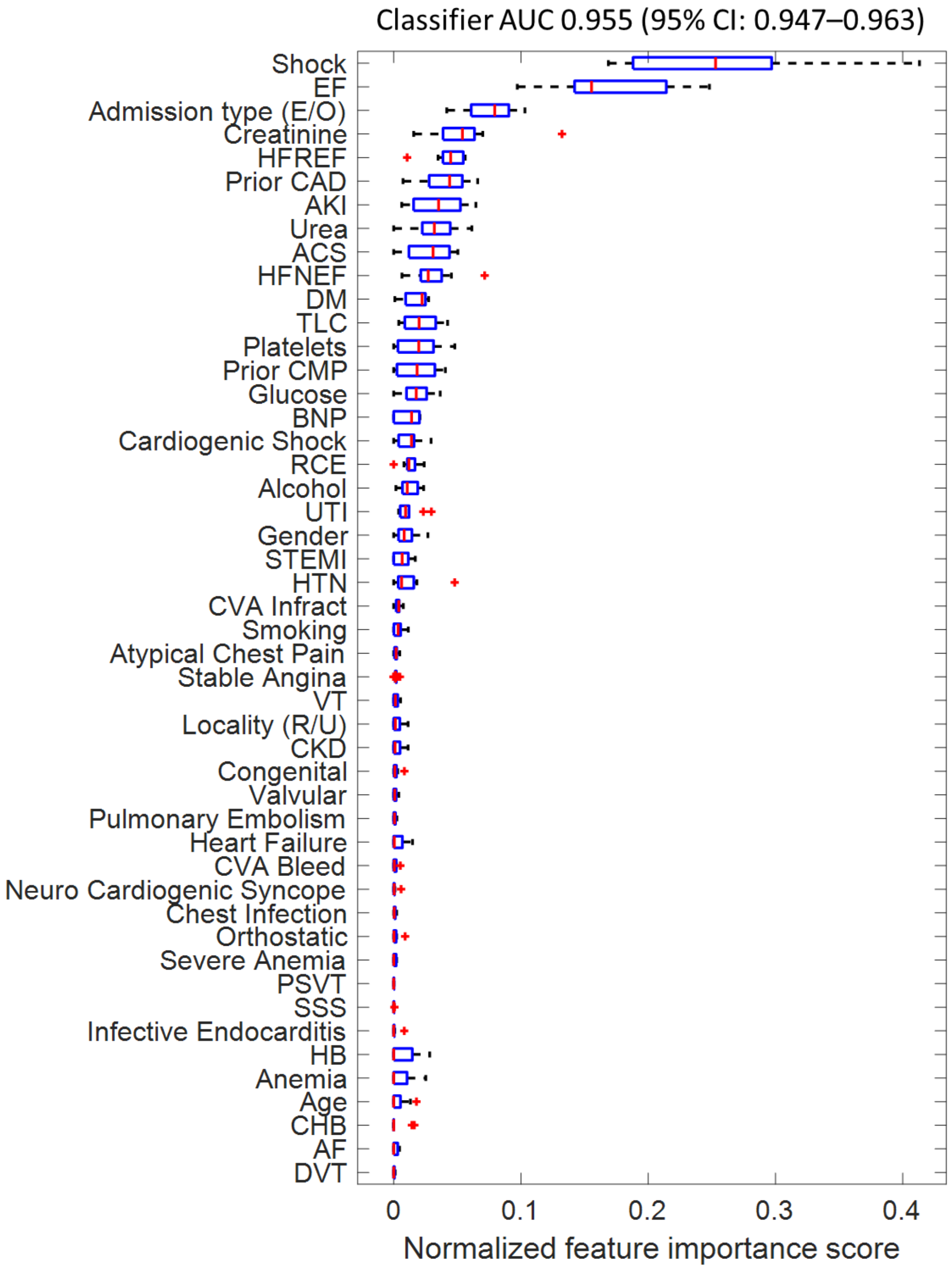

Feature importance of mortality classifier using feature set-1 (FS1) as input

Figure S1B

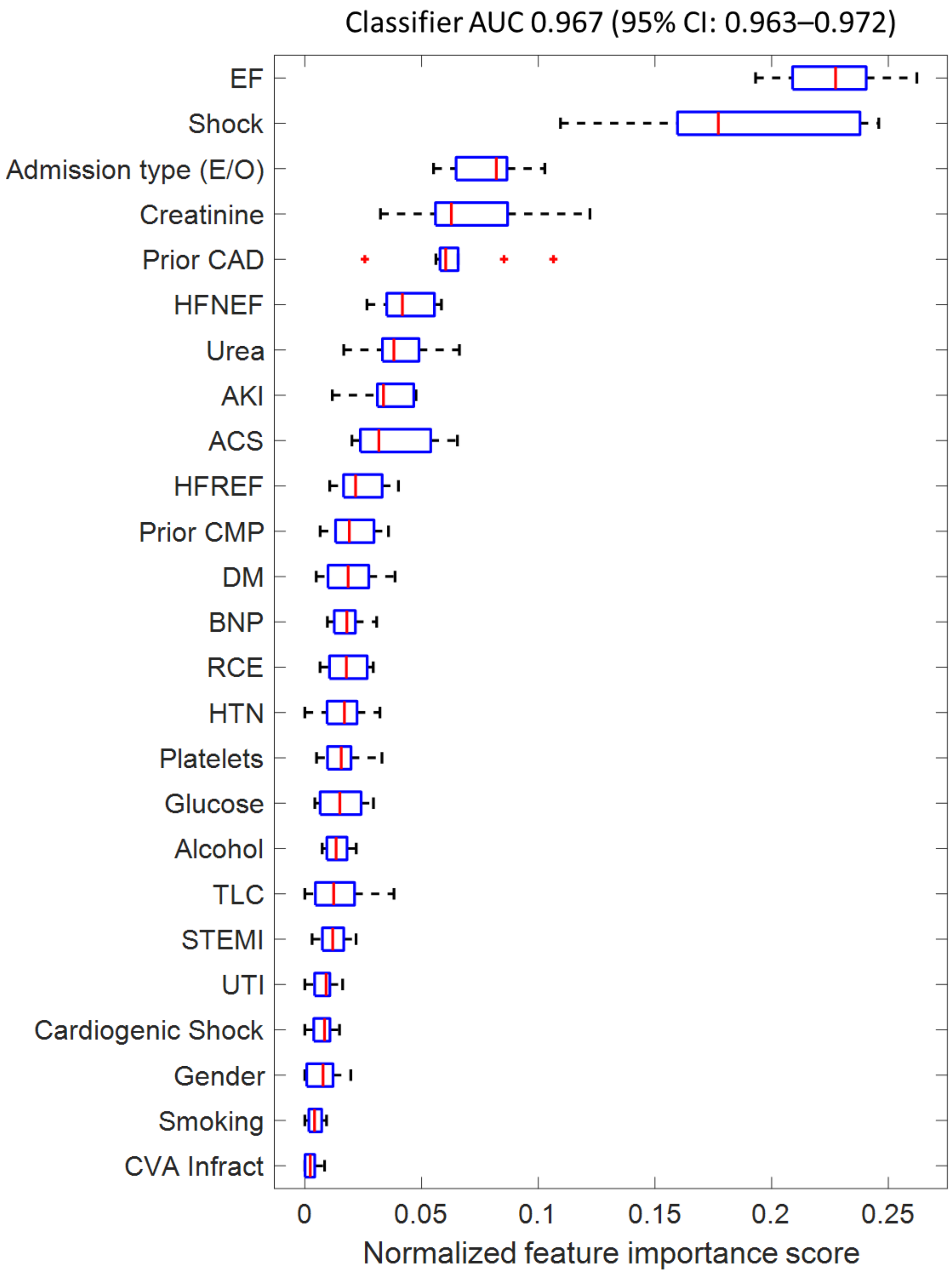

Feature importance of mortality classifier using feature set-2 (FS2) as input

Figure S1C

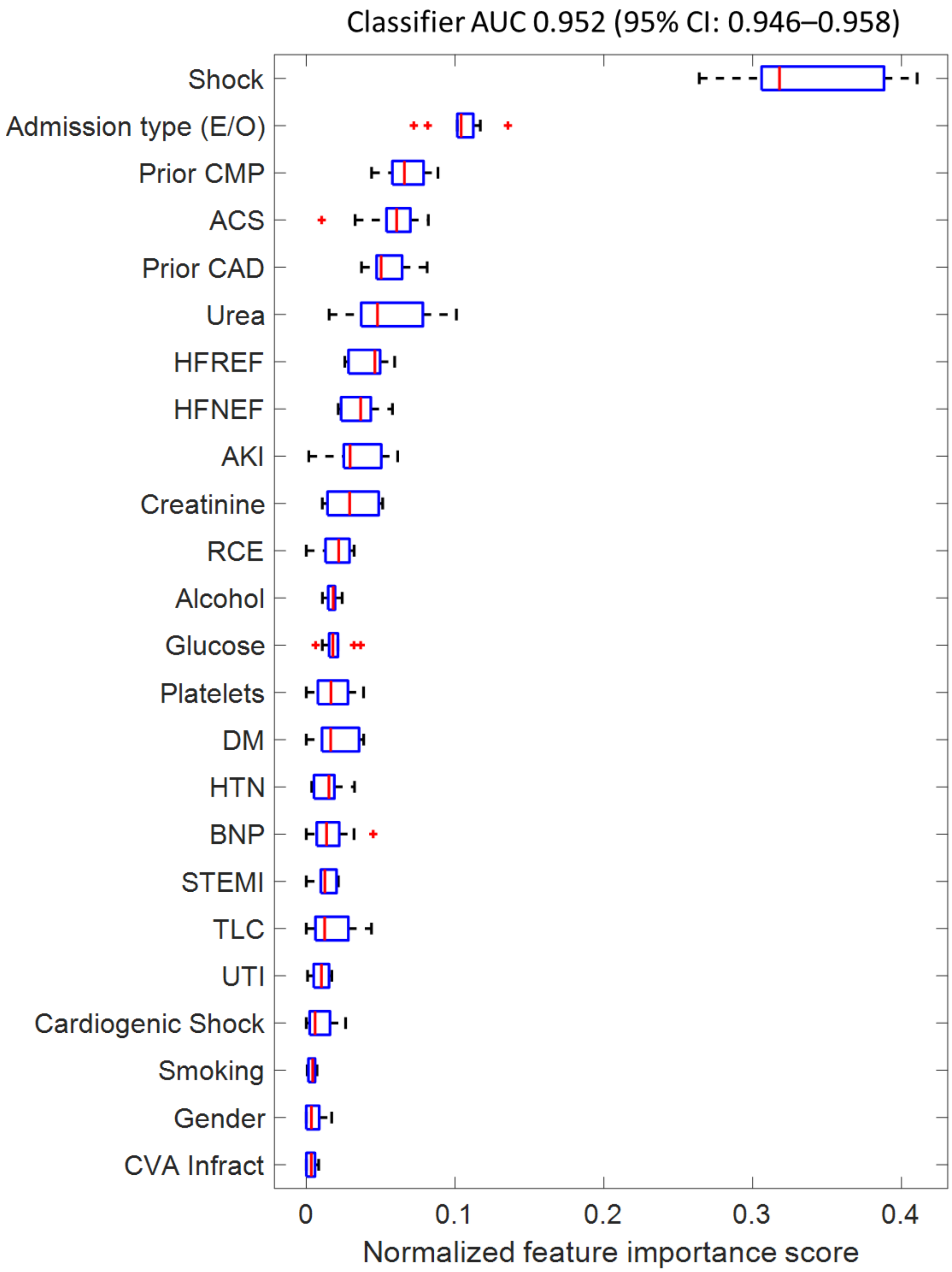

Feature importance of mortality classifier using feature set-3 (FS3) as input

Figure S1D

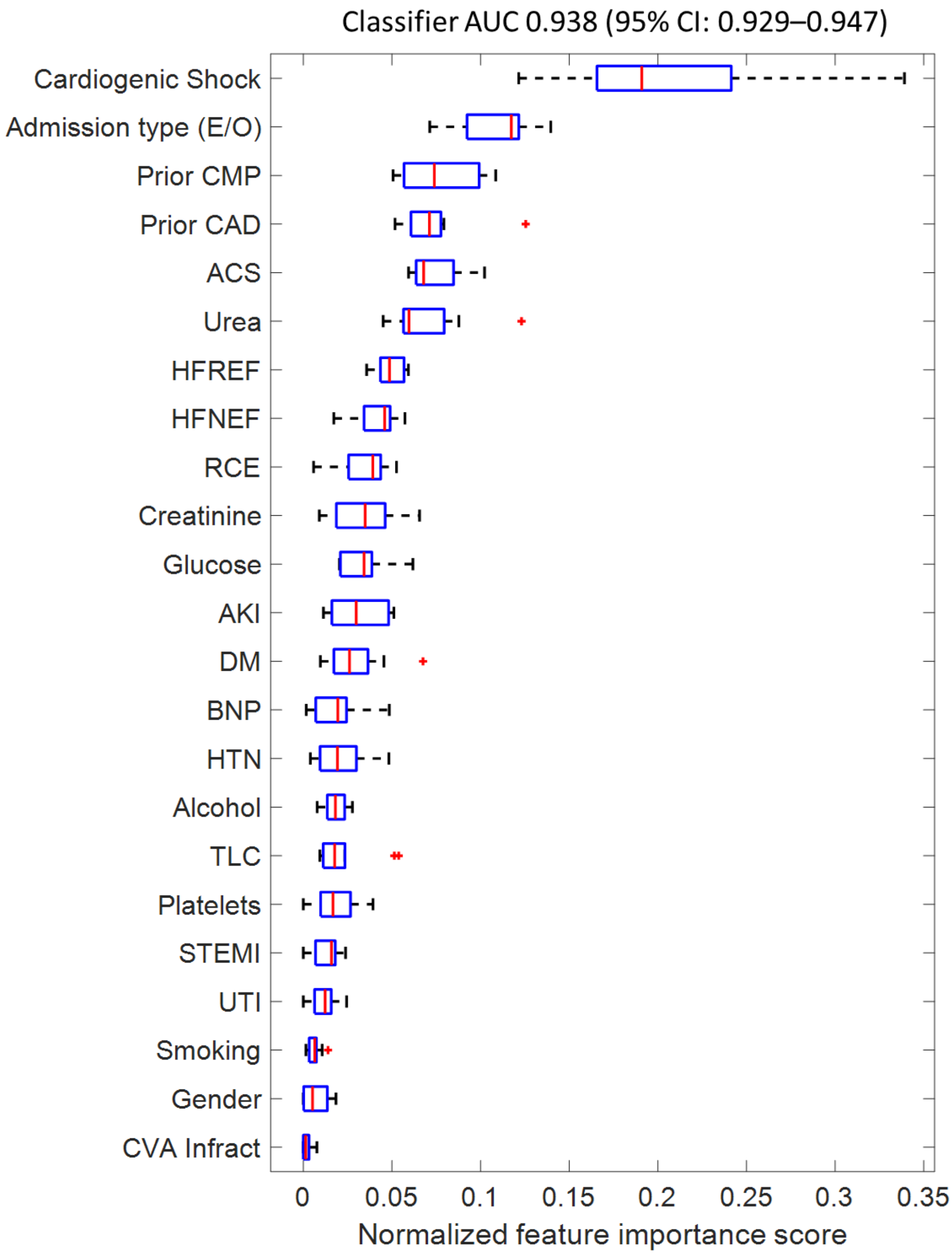

Feature importance of mortality classifier using feature set-4 (FS4) as input

Figure S1E

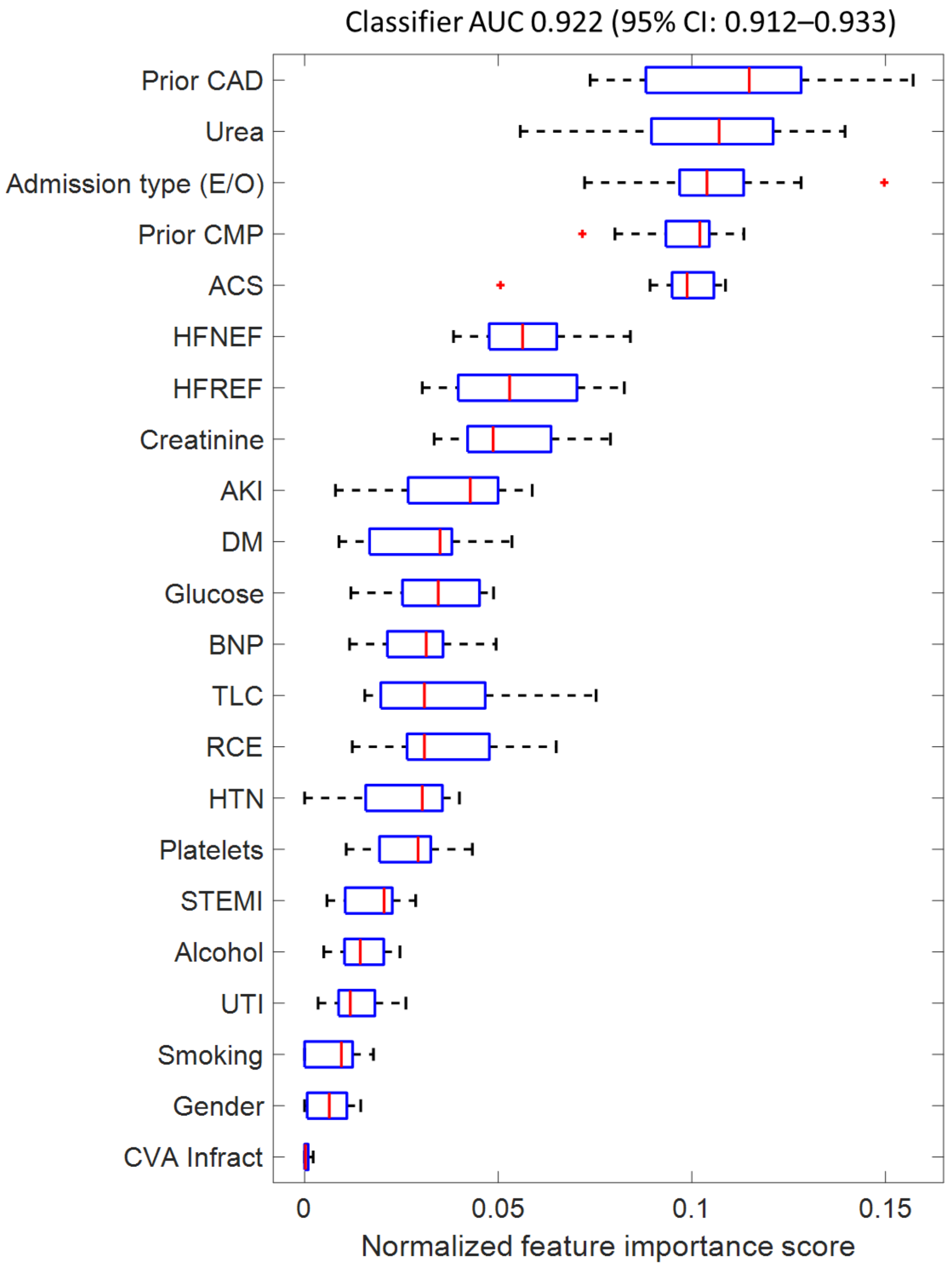

Feature importance of mortality classifier using feature set-5 (FS5) as input

Figure S1F

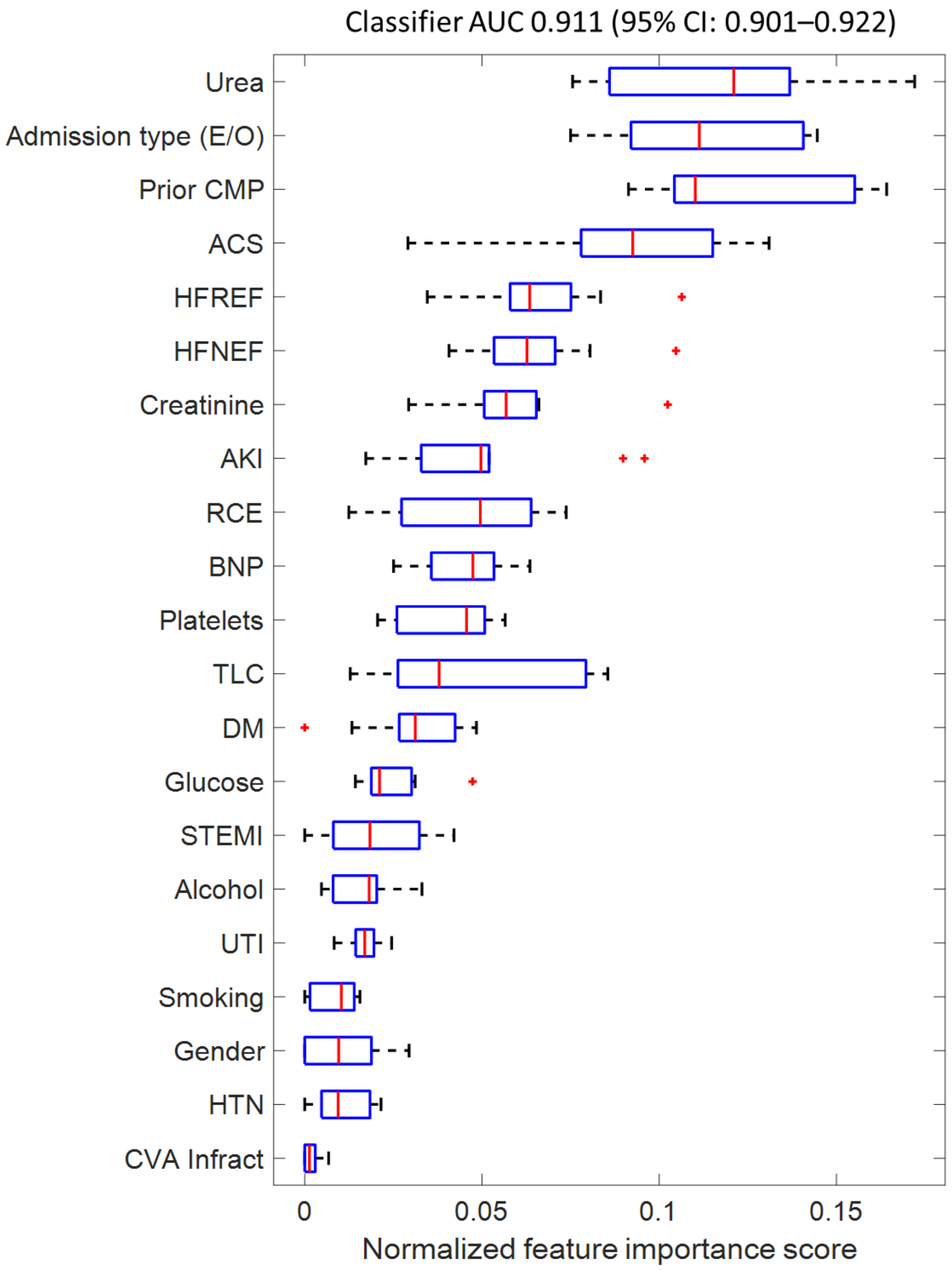

Feature importance of mortality classifier using feature set-6 (FS6) as input

Figure S1G

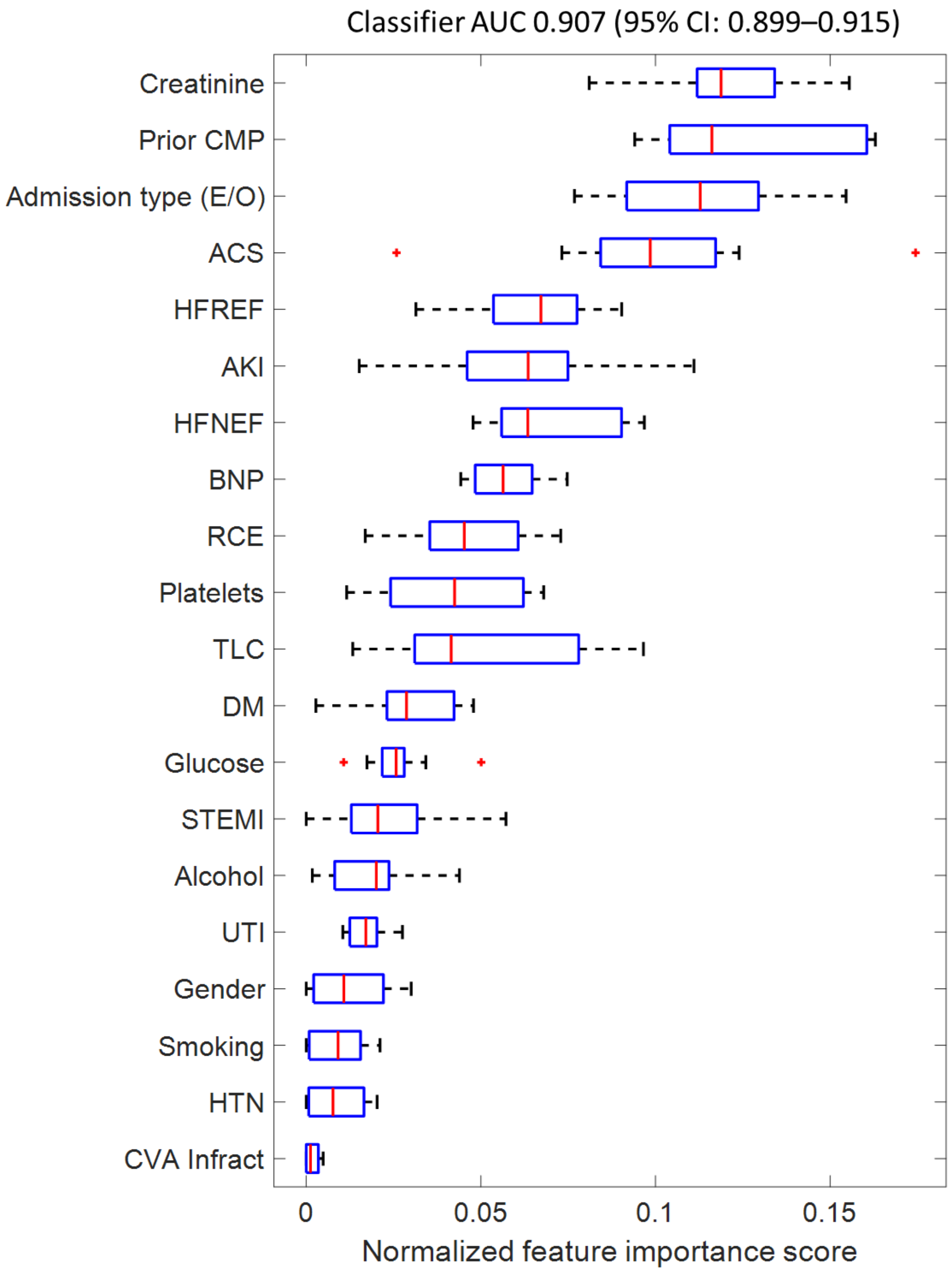

Feature importance of mortality classifier using feature set-7 (FS7) as input

Online Supplement Figure S1. Feature importance scores for predicting mortality using (A) FS1; (B) FS2; (C) FS3; (D) FS4; (E) FS5; (F) FS6; (G) FS7. Abbreviations: Admission type (E/O): emergency/outpatient; Locality (R/U): rural/urban; DM: diabetes mellitus, HTN: hypertension, CAD: coronary artery disease, CMP: cardiomyopathy, CKD: chronic kidney disease; HB: hemoglobin, TLC: total lymphocyte count, BNP: brain natriuretic peptide, RCE: raised cardiac enzymes and EF: ejection fraction.

Figure S2A

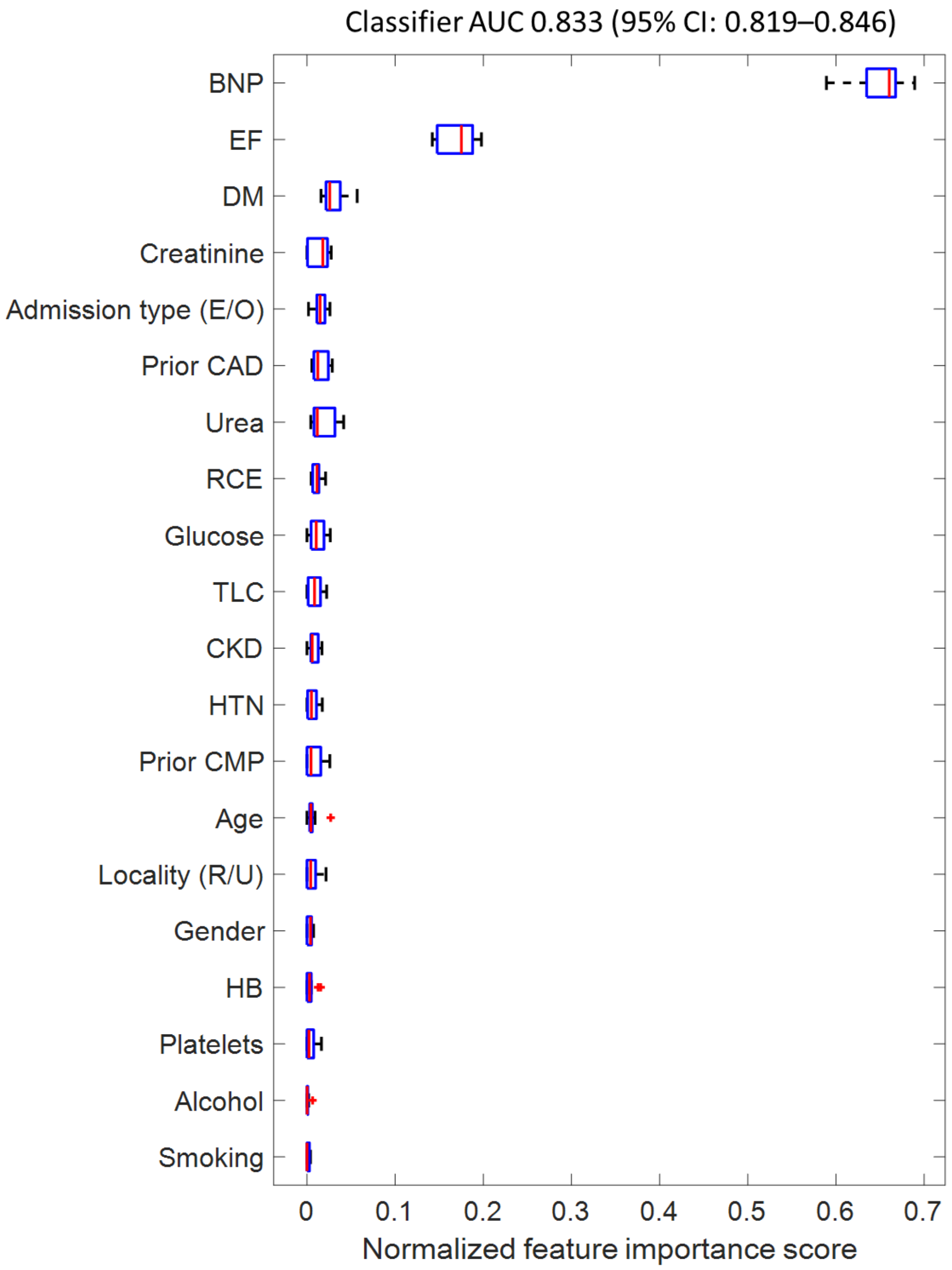

Feature importance of heart failure classifier using feature set-1 (FS1) as input

Figure S2B

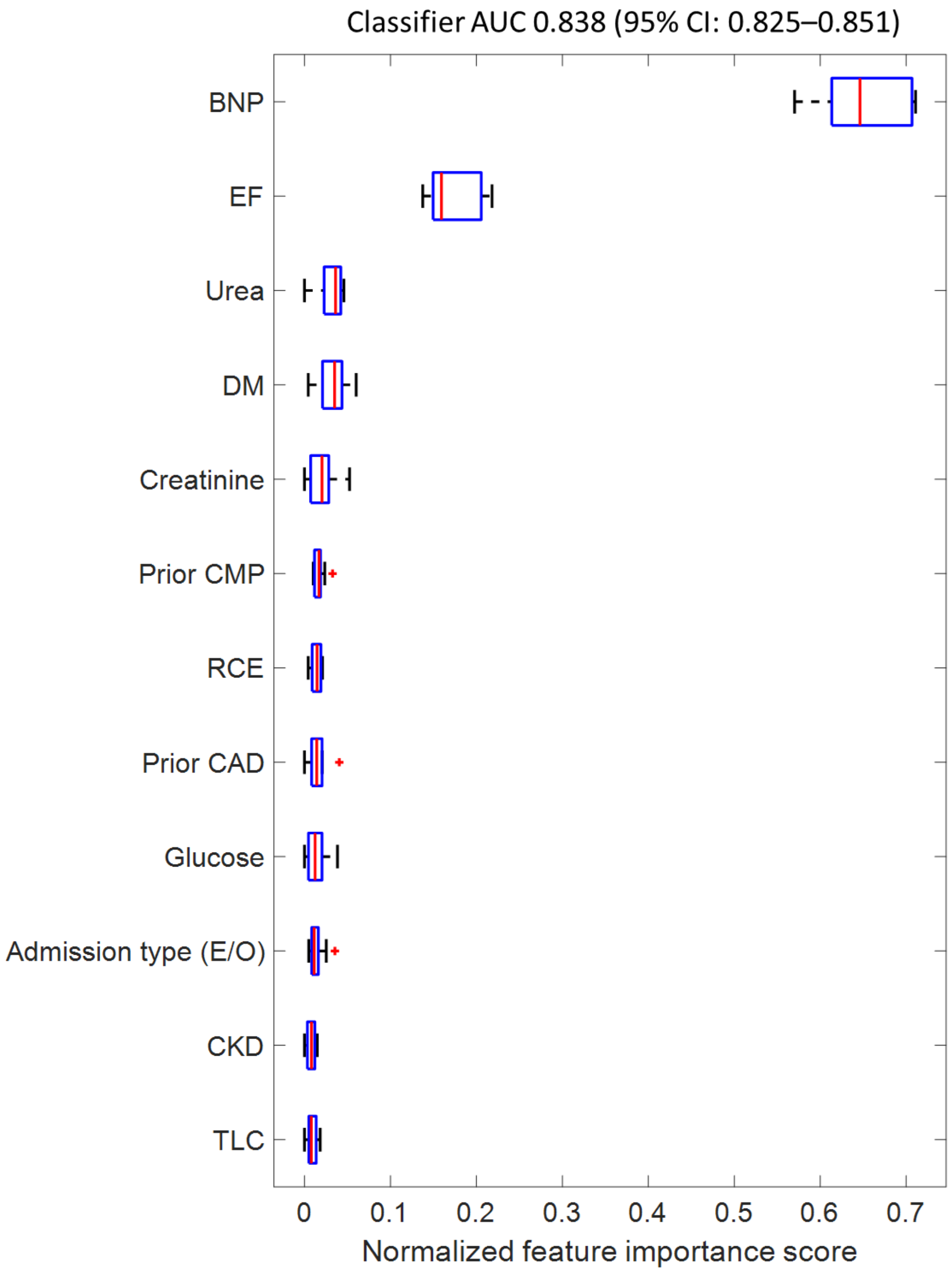

Feature importance of heart failure classifier using feature set-2 (FS2) as input

Figure S2C

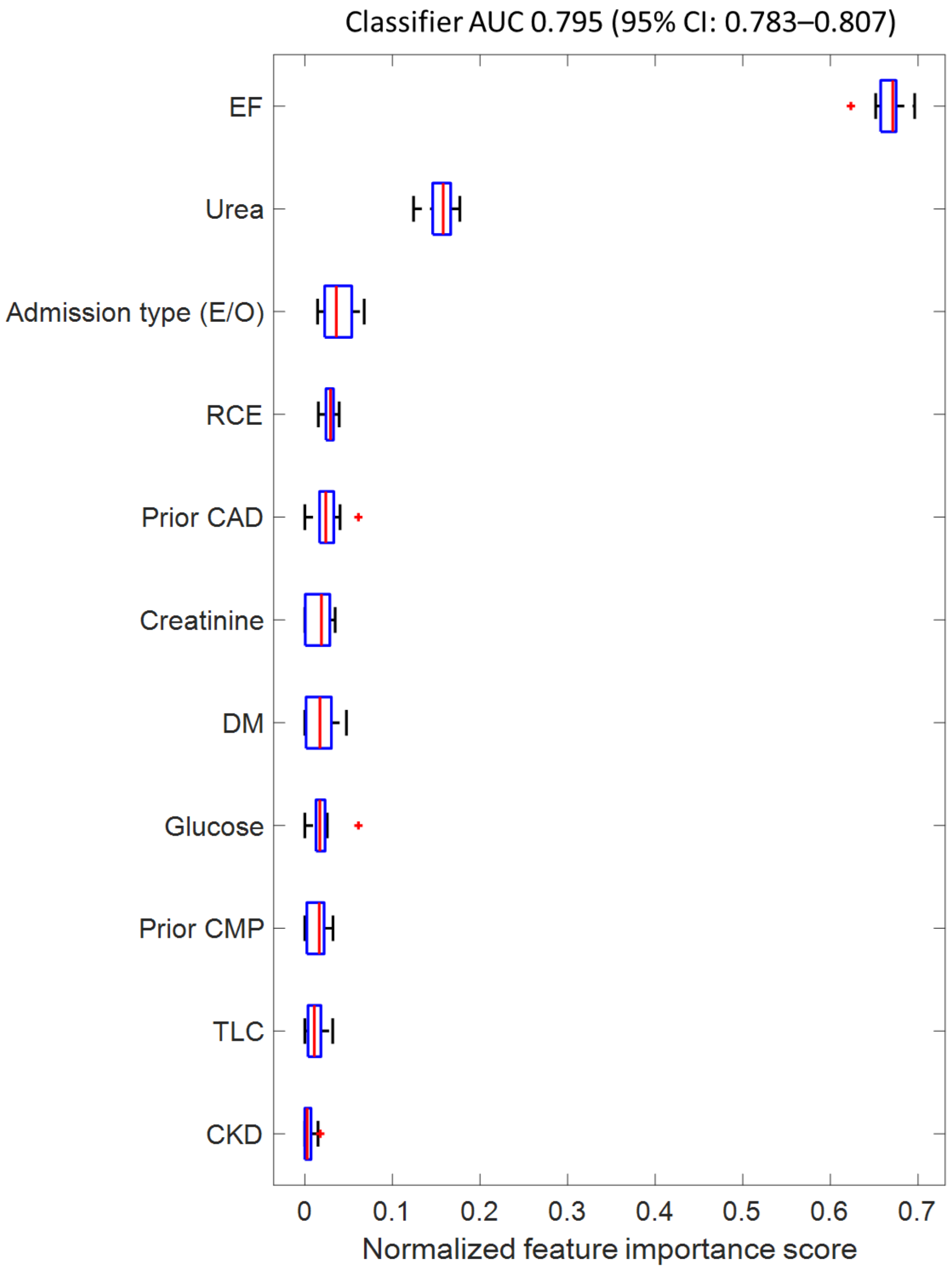

Feature importance of heart failure classifier using feature set-3 (FS3) as input

Figure S2D

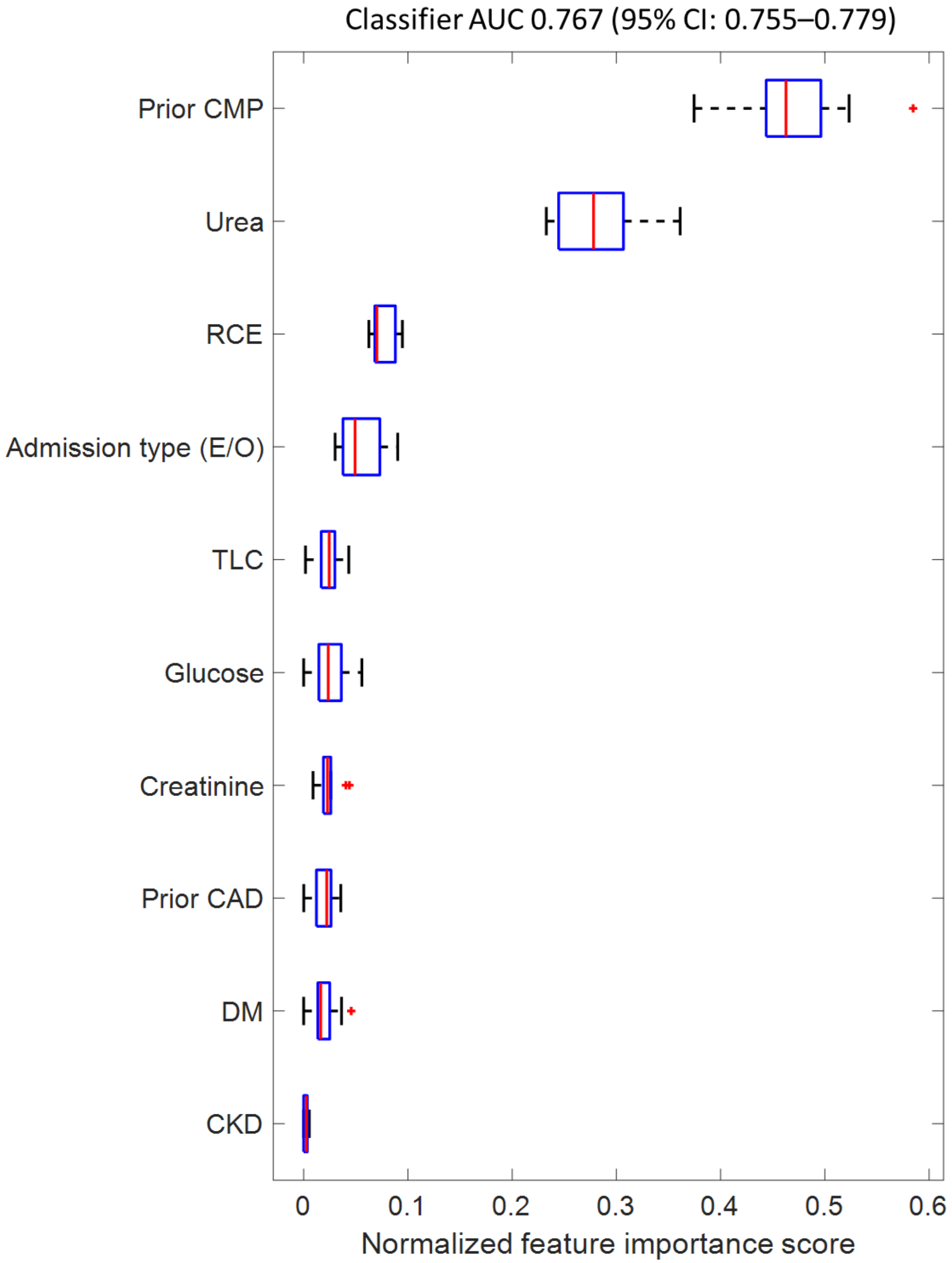

Feature importance of heart failure classifier using feature set-4 (FS4) as input

Figure S2E

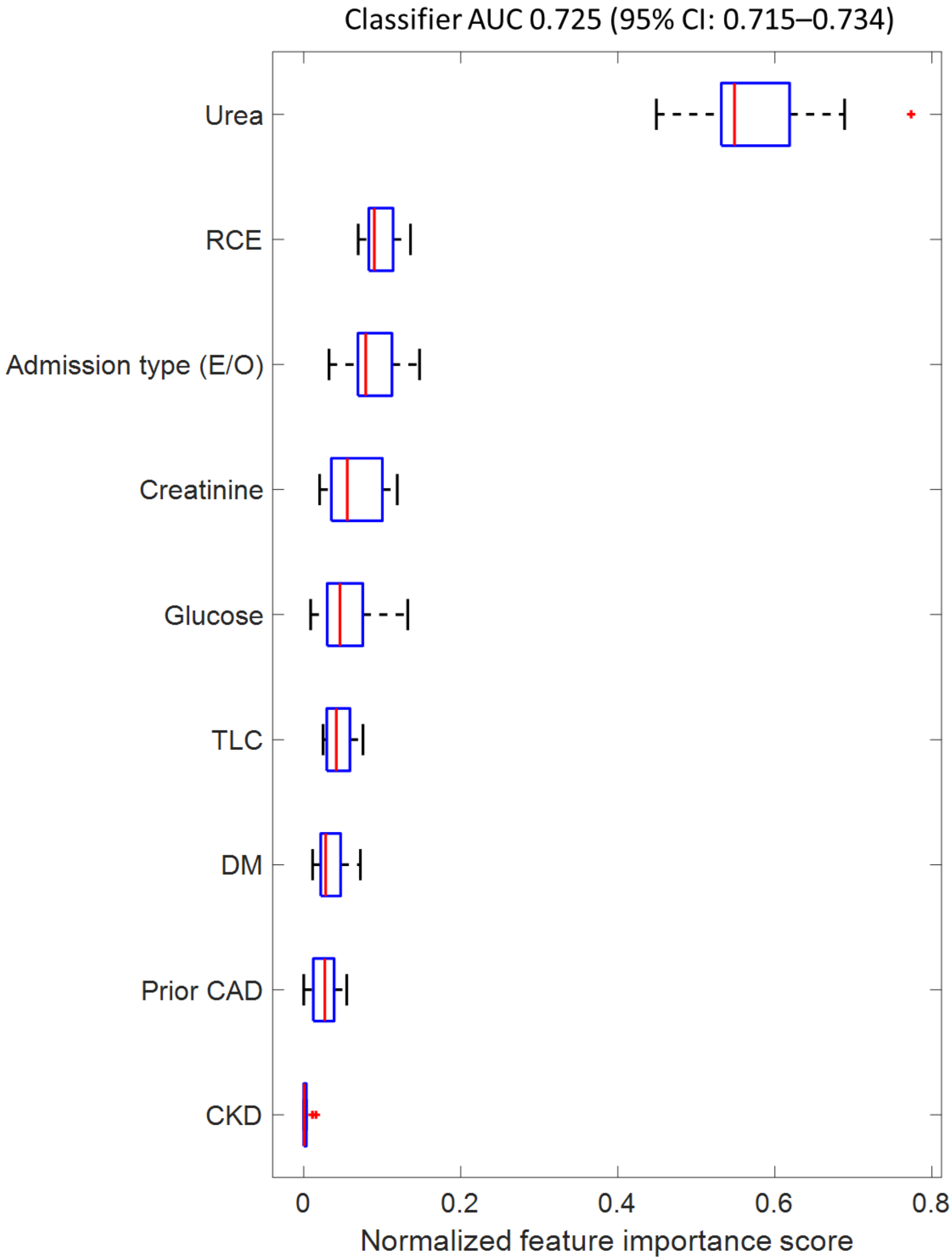

Feature importance of heart failure classifier using feature set-5 (FS5) as input

Figure S2F

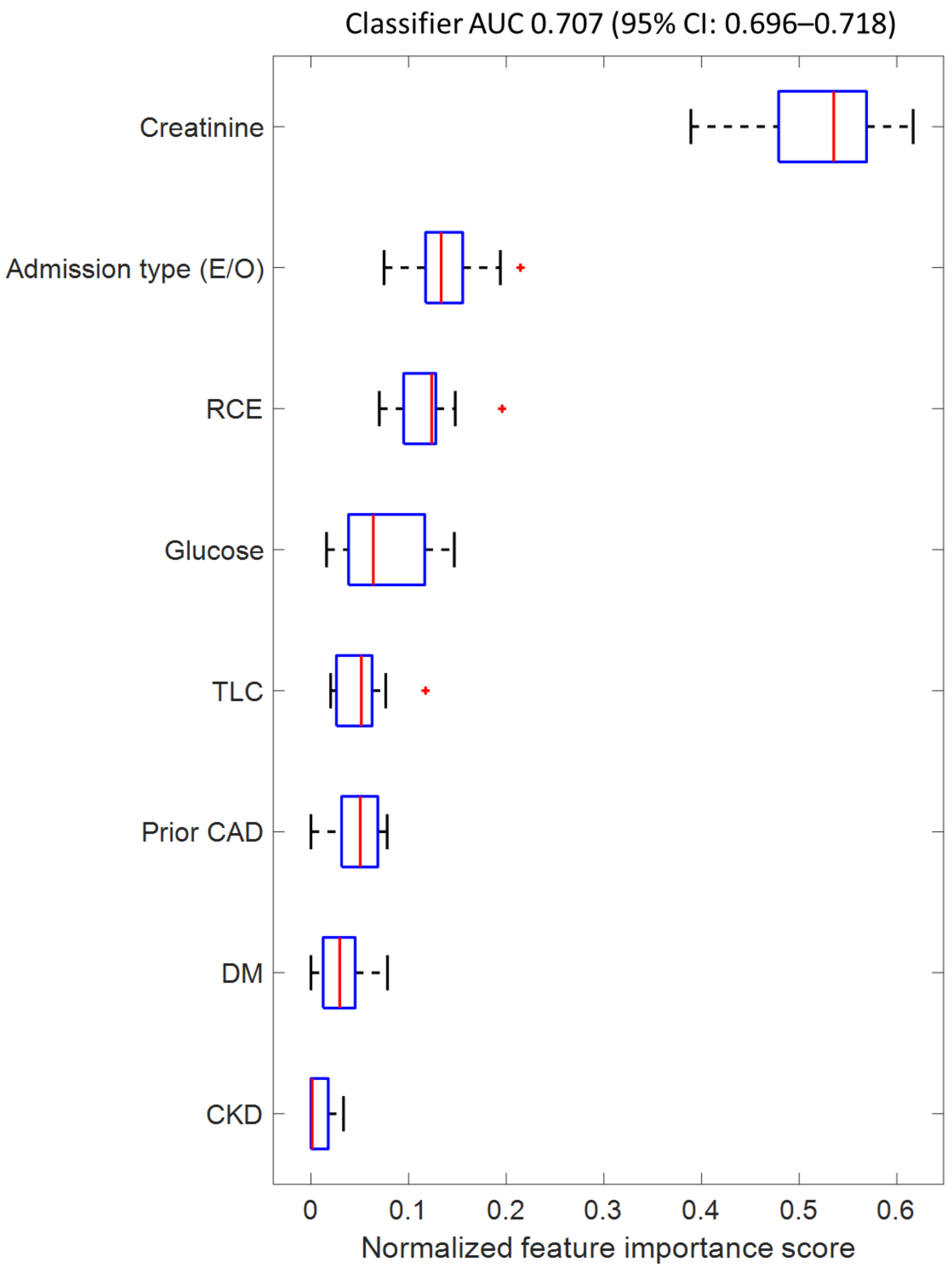

Feature importance of heart failure classifier using feature set-6 (FS6) as input

Figure S2G

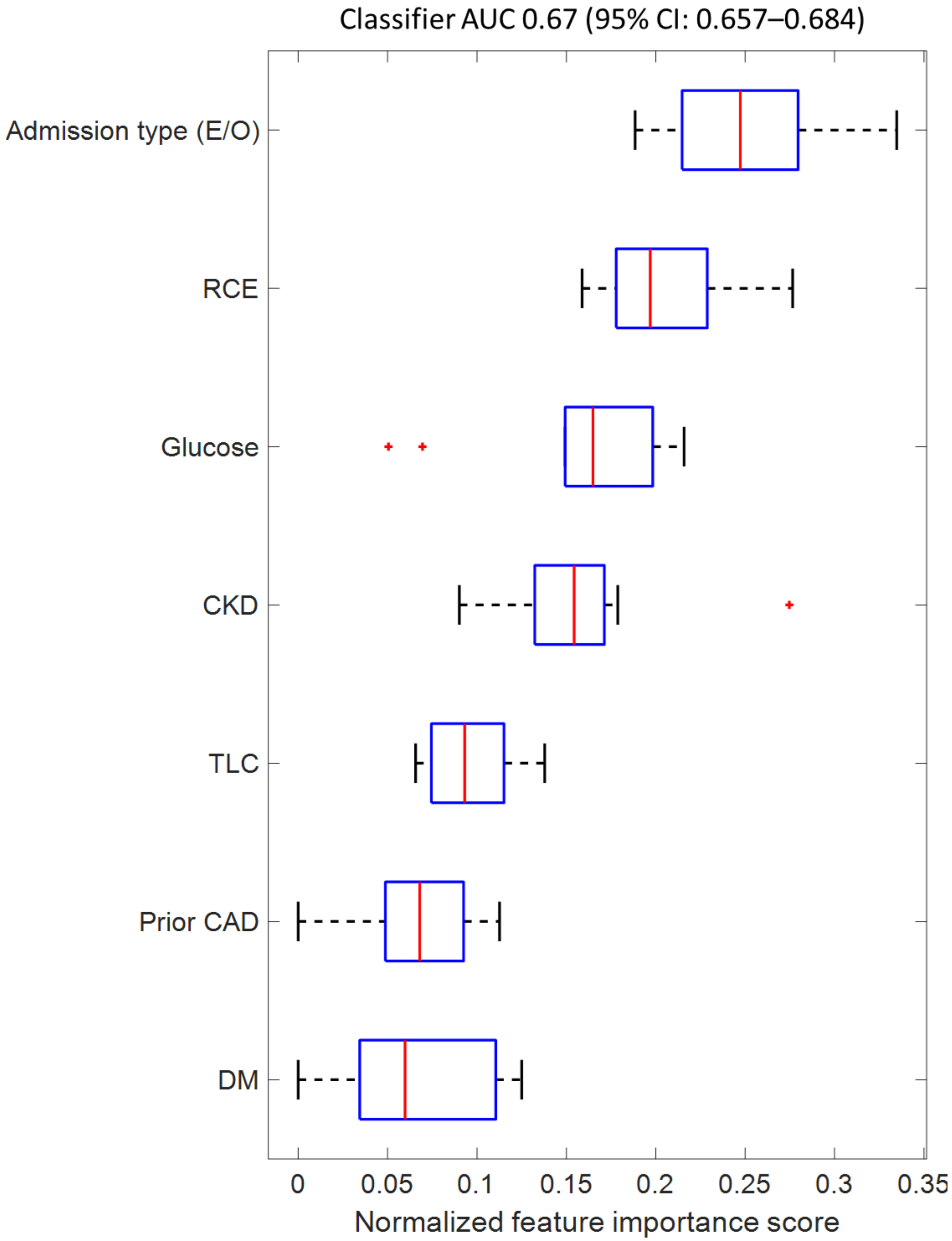

Feature importance of heart failure classifier using feature set-7 (FS7) as input

Online Supplement Figure S2. Feature importance scores for predicting heart failure using (A) FS1; (B) FS2; (C) FS3; (D) FS4; (E) FS5; (F) FS6; (G) FS7.

Figure S3A

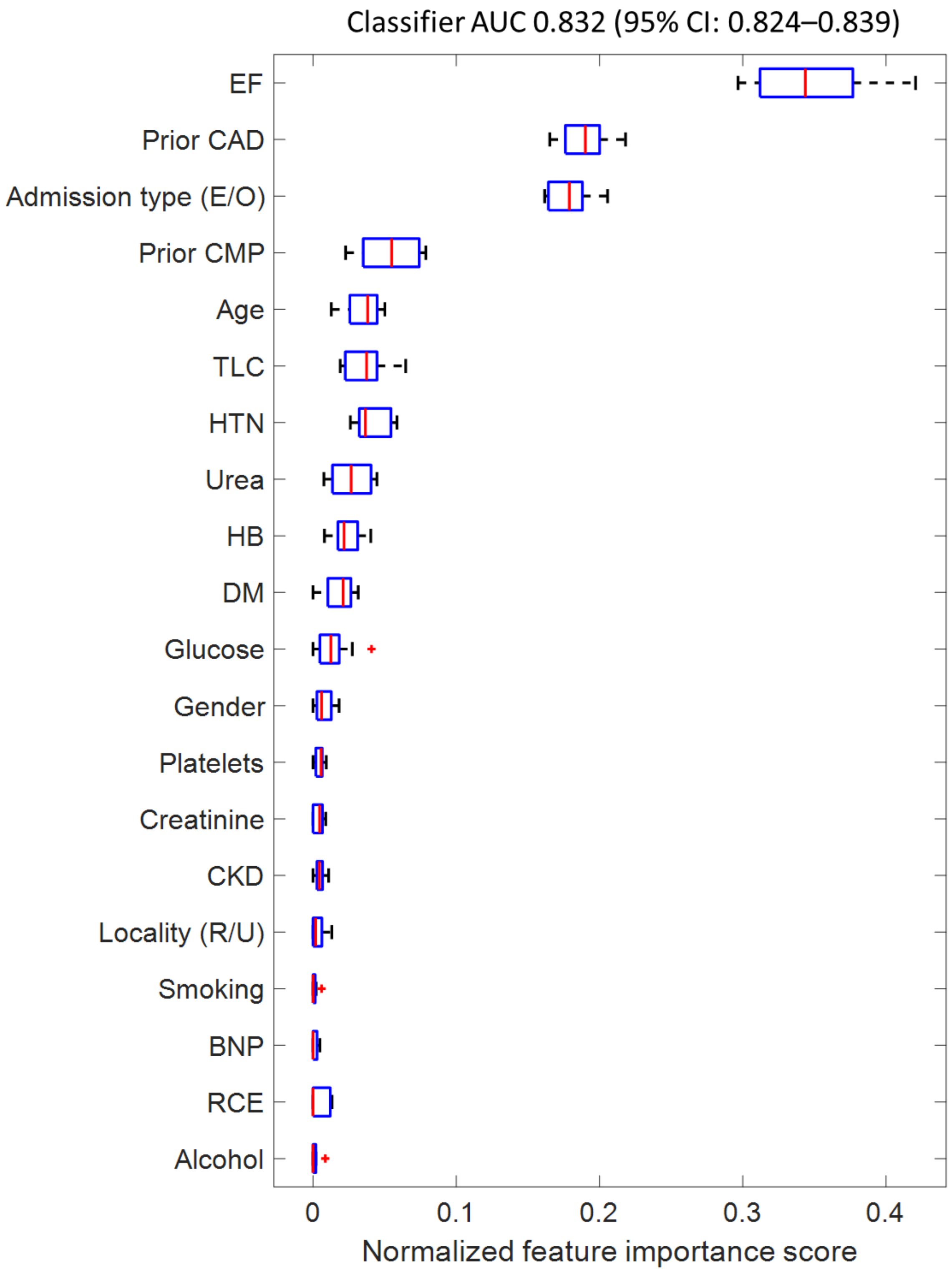

Feature importance of STEMI classifier using feature set-1 (FS1) as input

Figure S3B

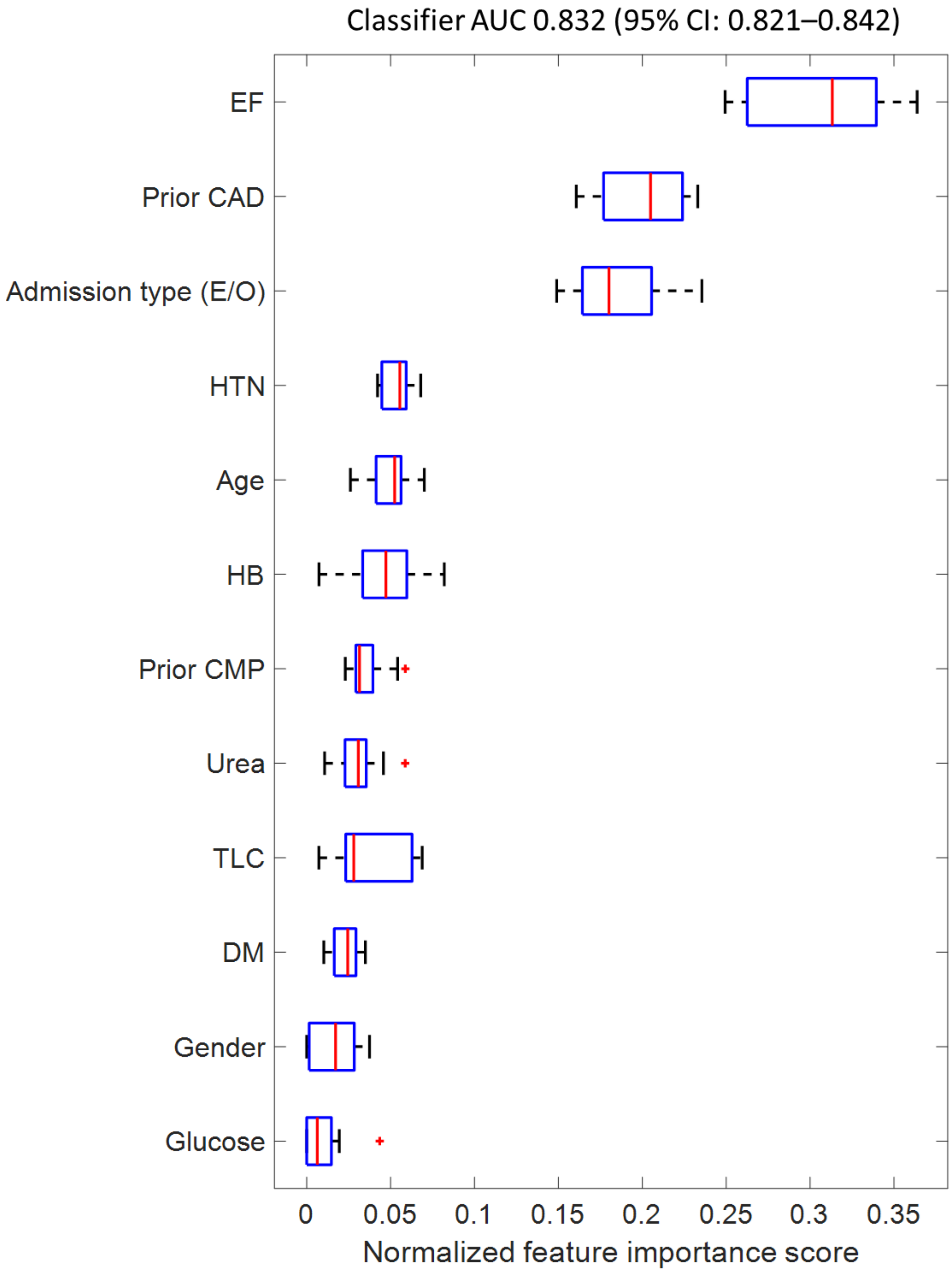

Feature importance of STEMI classifier using feature set-2 (FS2) as input

Figure S3C

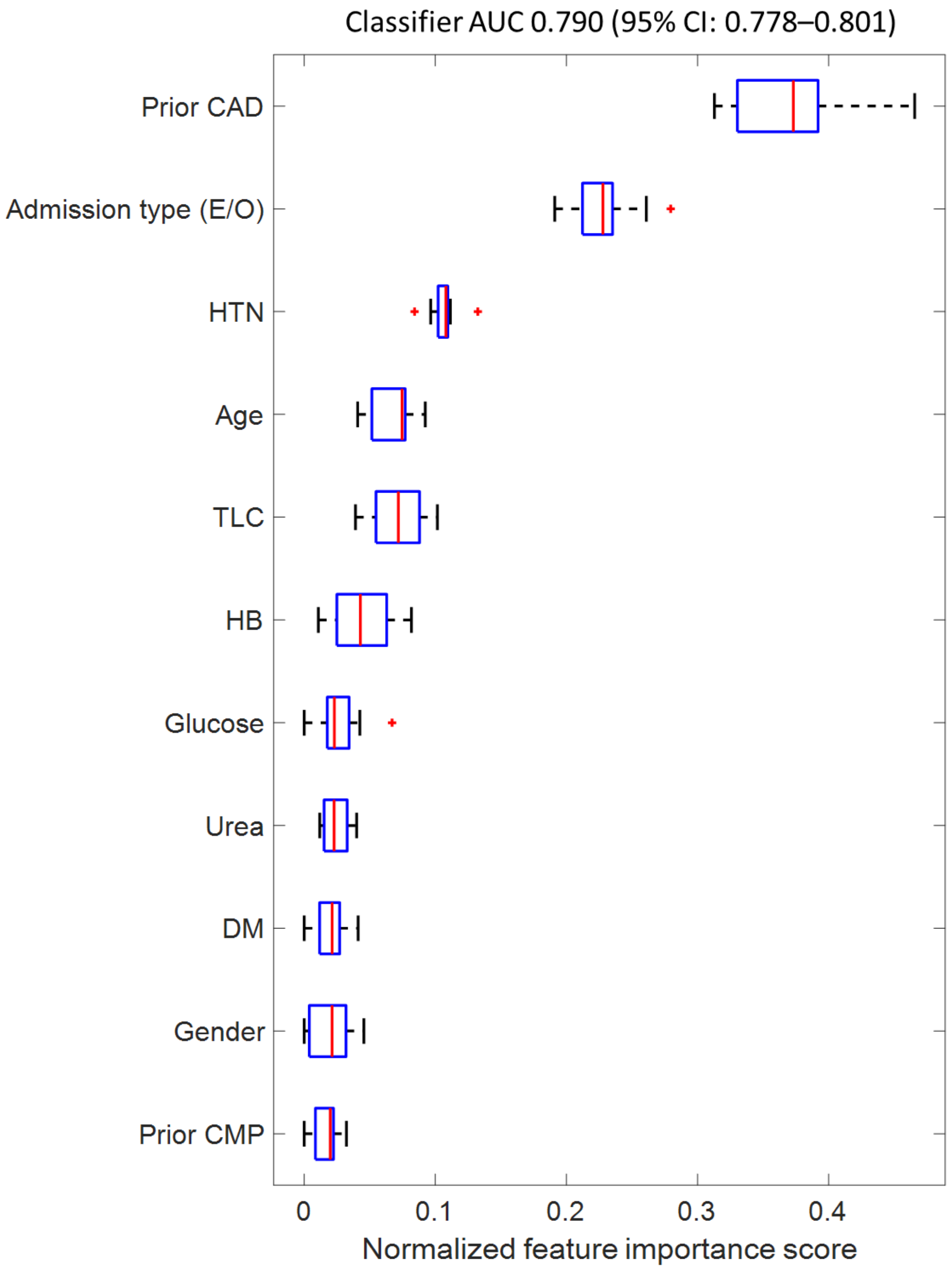

Feature importance of STEMI classifier using feature set-3 (FS3) as input

Figure S3D

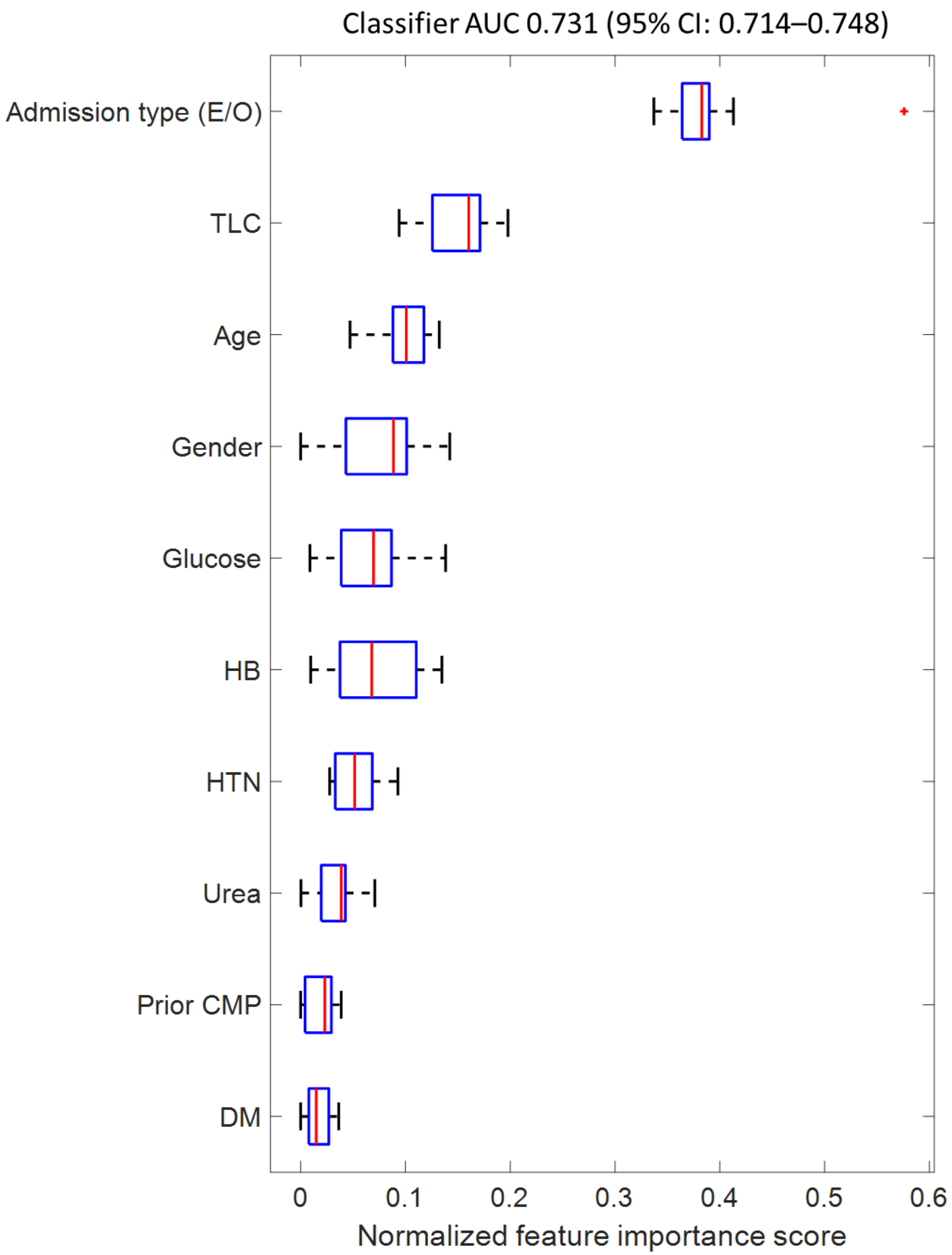

Feature importance of STEMI classifier using feature set-4 (FS4) as input

Figure S3E

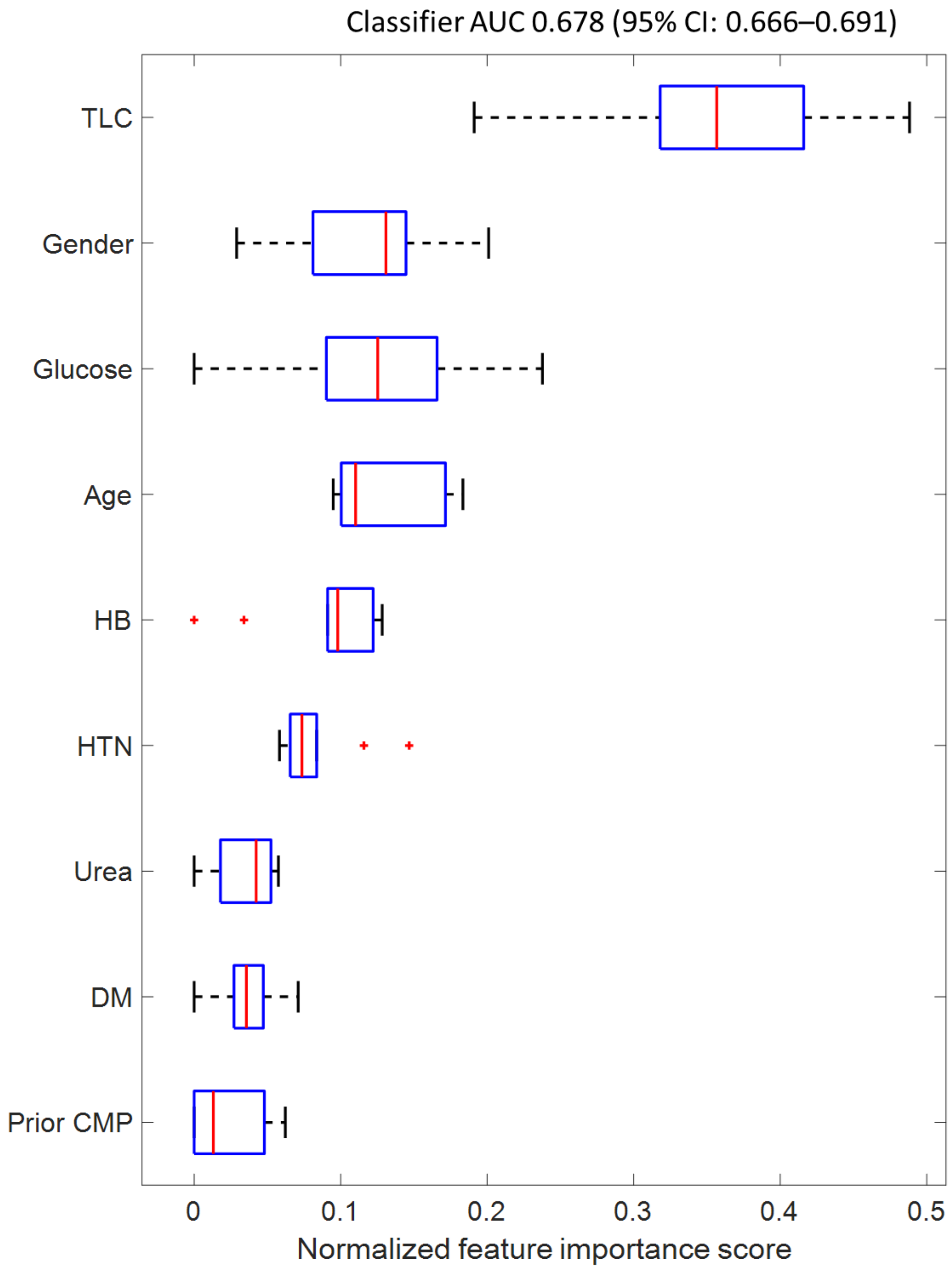

Feature importance of STEMI classifier using feature set-5 (FS5) as input

Figure S3F

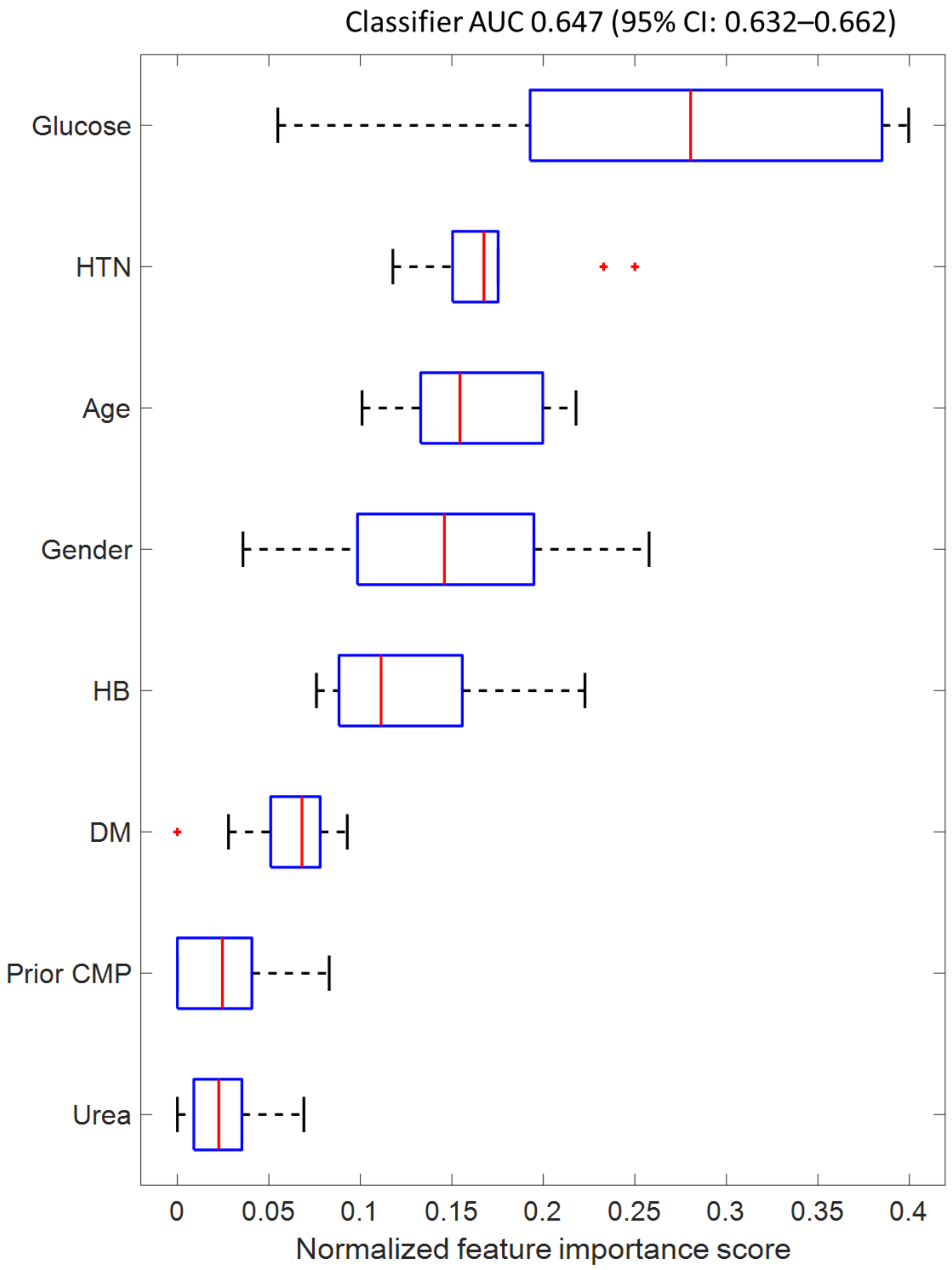

Feature importance of STEMI classifier using feature set-6 (FS6) as input

Figure S3G

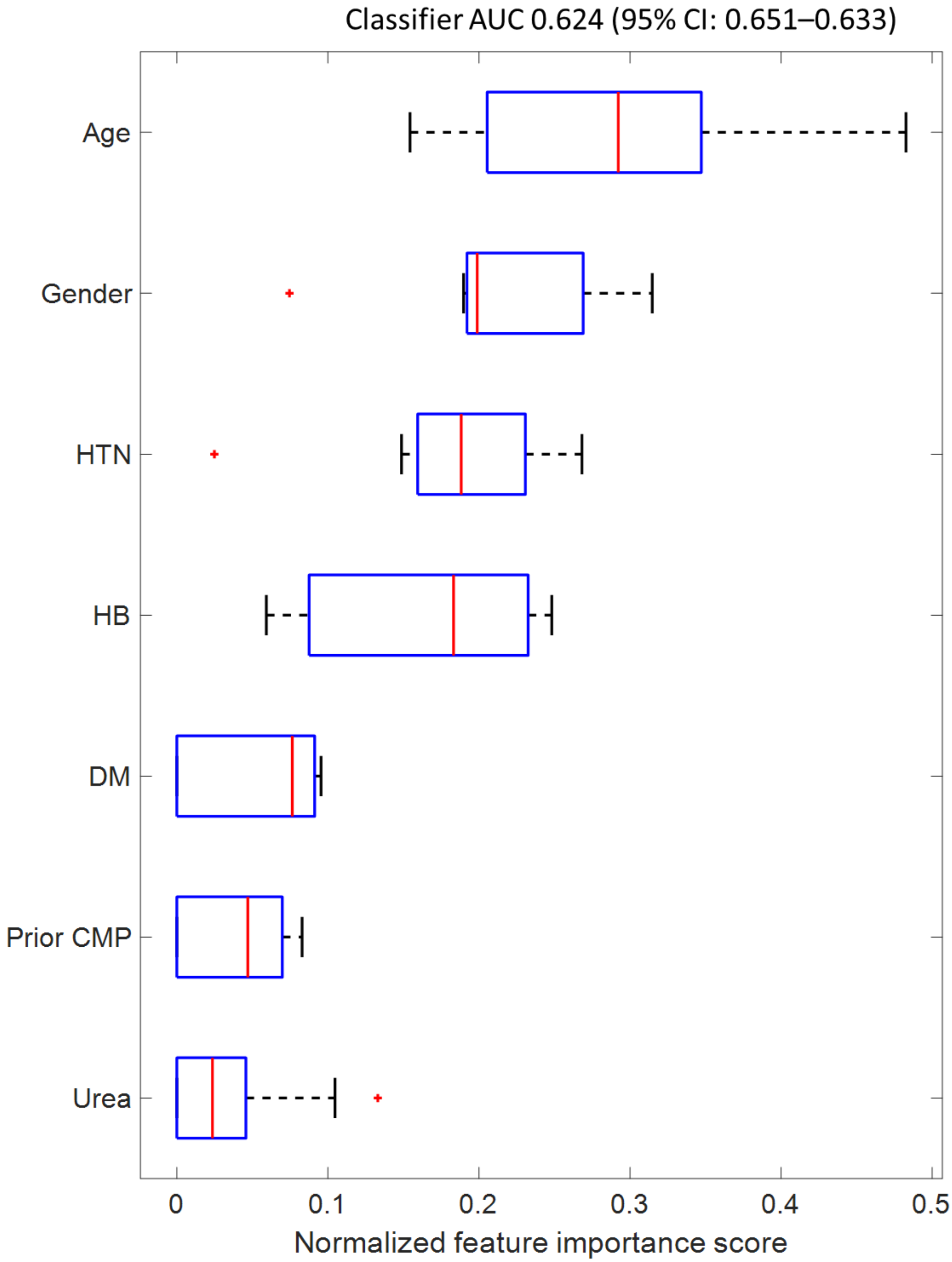

Feature importance of STEMI classifier using feature set-7 (FS7) as input

Online Supplement Figure S3. Feature importance scores for predicting of ST-segment elevation myocardial infarction using (A) FS1; (B) FS2; (C) FS3; (D) FS4; (E) FS5; (F) FS6; (G) FS7.

Figure S4A

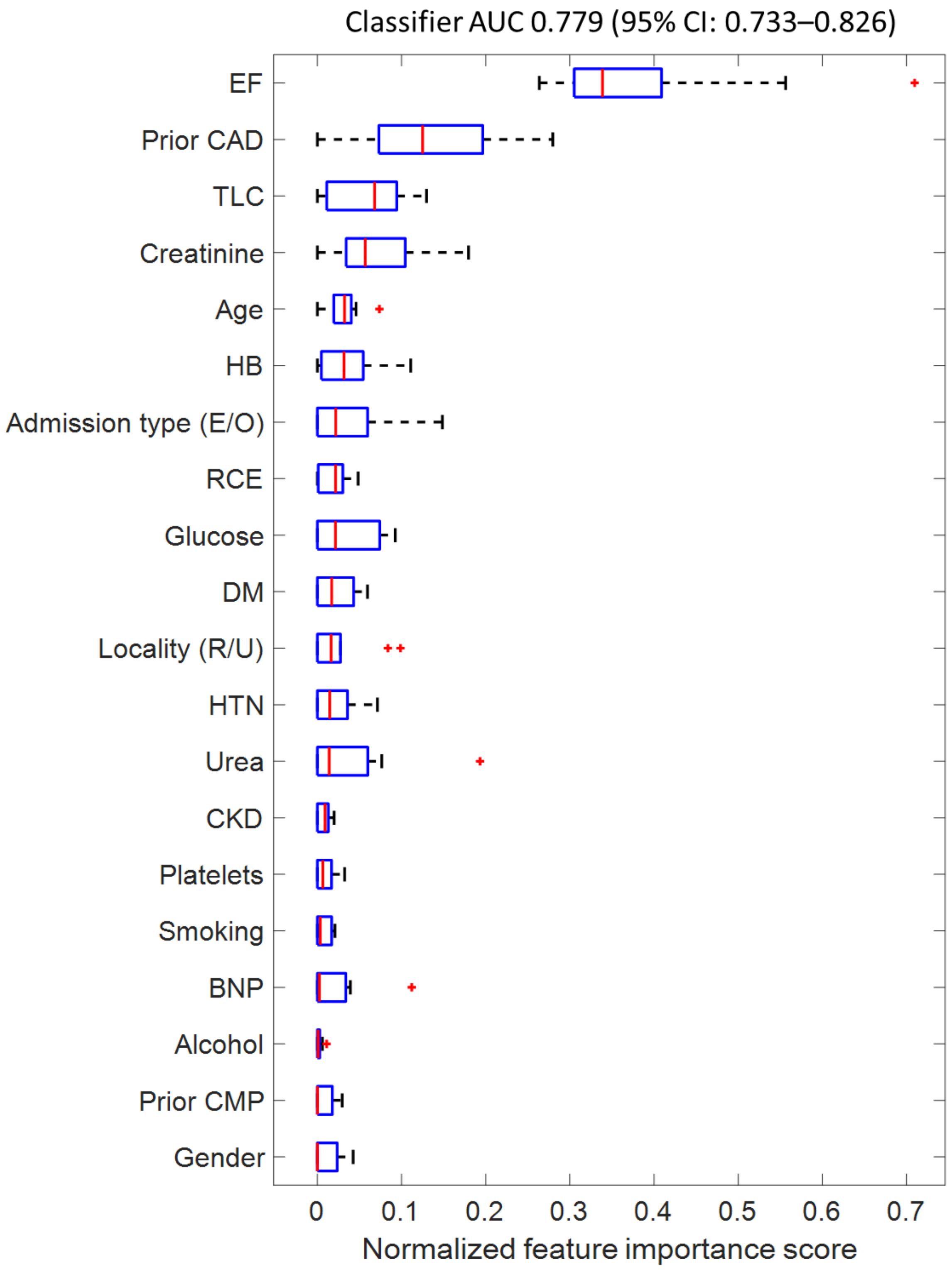

Feature importance of pulmonary embolism classifier using feature set-1 (FS1) as input

Figure S4B

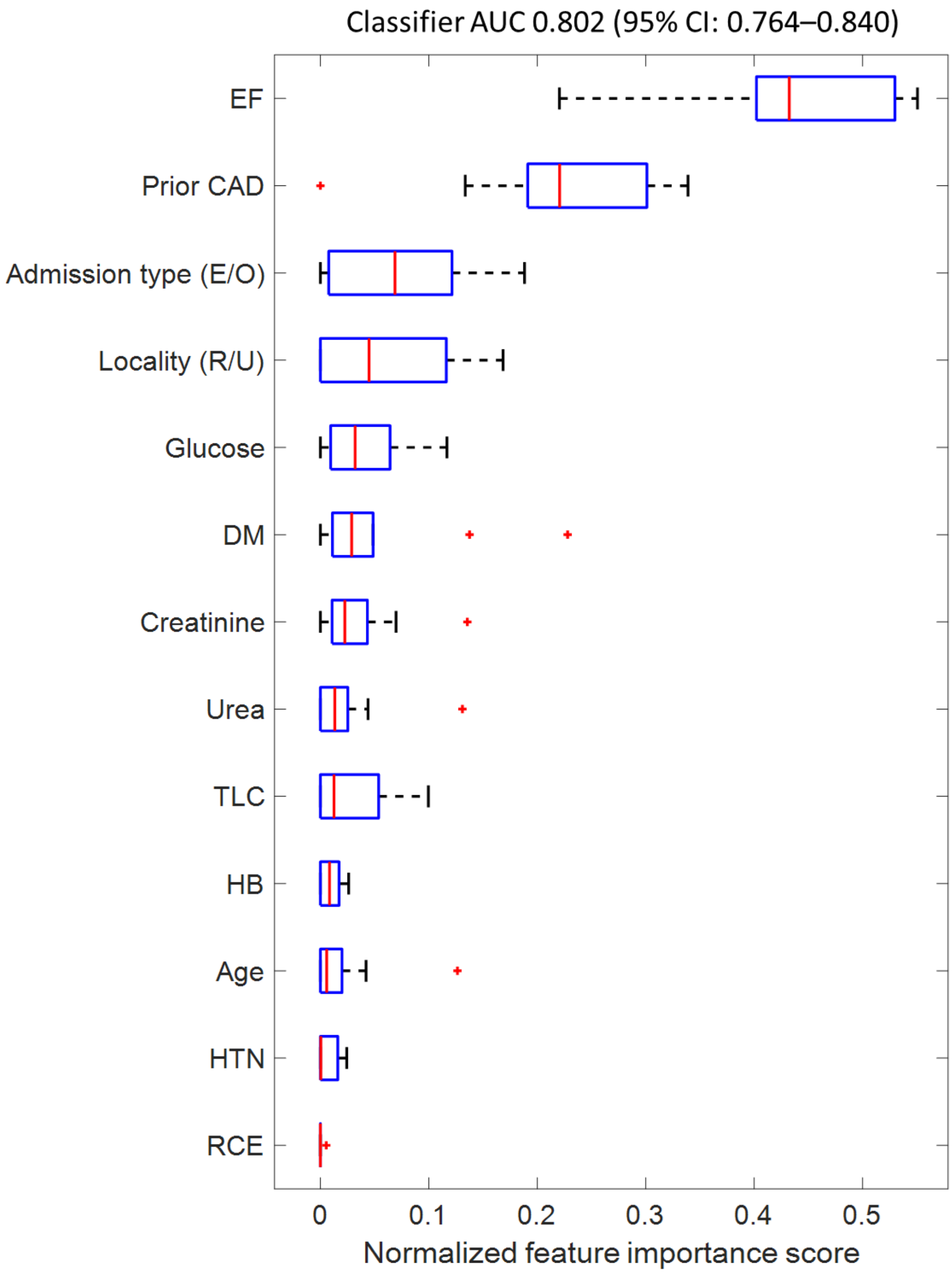

Feature importance of pulmonary embolism classifier using feature set-2 (FS2) as input

Figure S4C

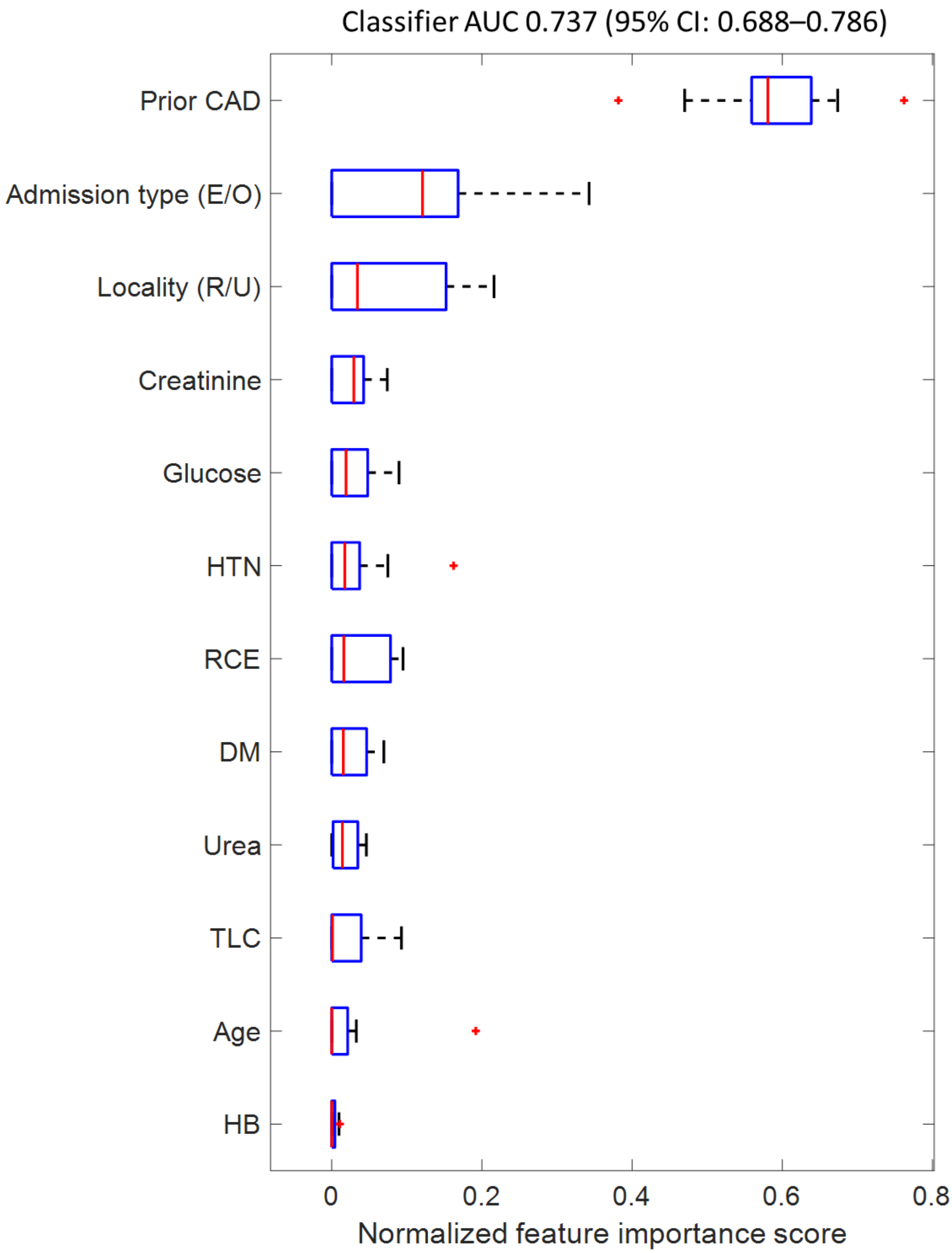

Feature importance of pulmonary embolism classifier using feature set-3 (FS3) as input

Figure S4D

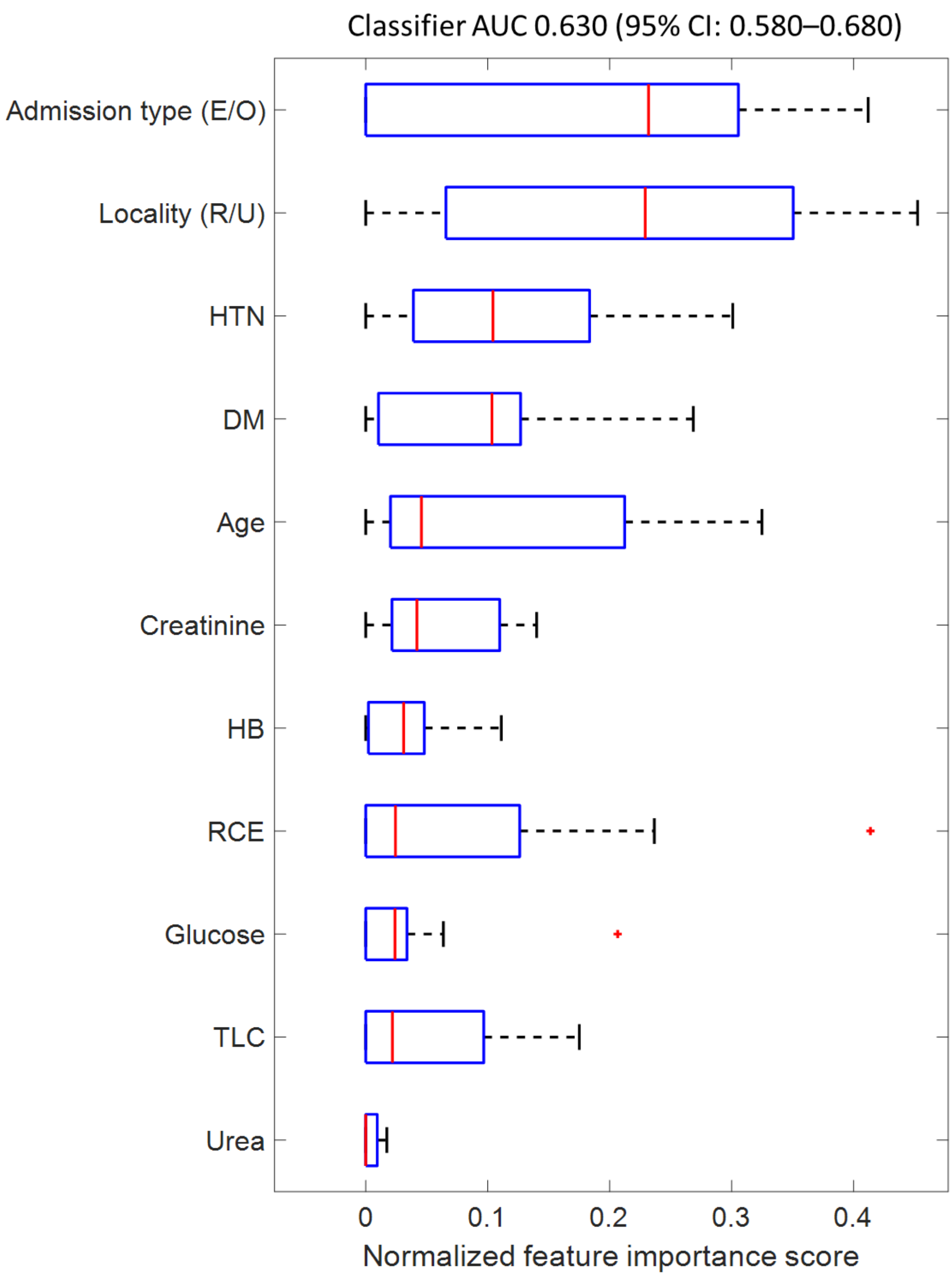

Feature importance of pulmonary embolism classifier using feature set-4 (FS4) as input

Figure S4E

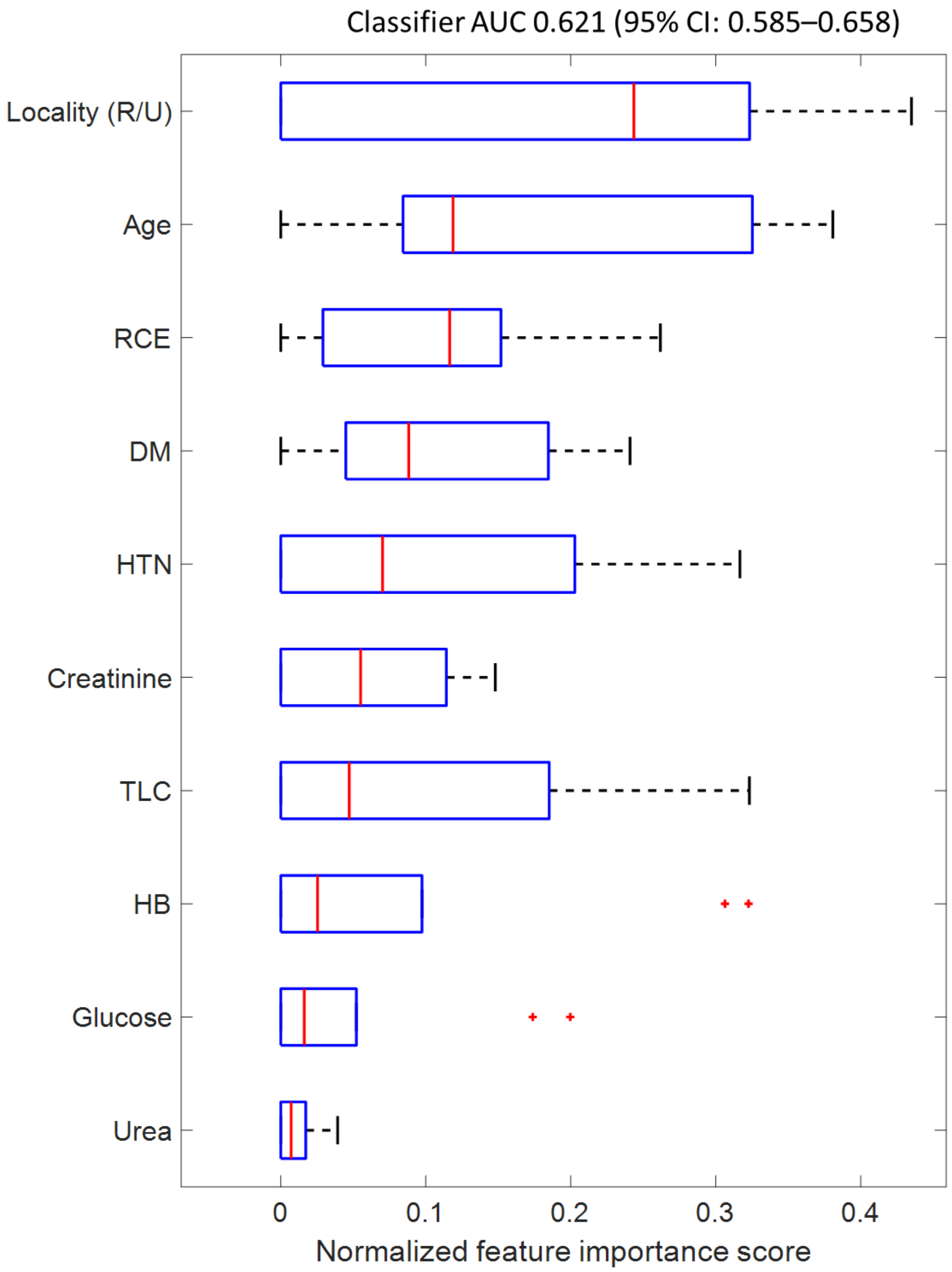

Feature importance of pulmonary embolism classifier using feature set-5 (FS5) as input

Figure S4F

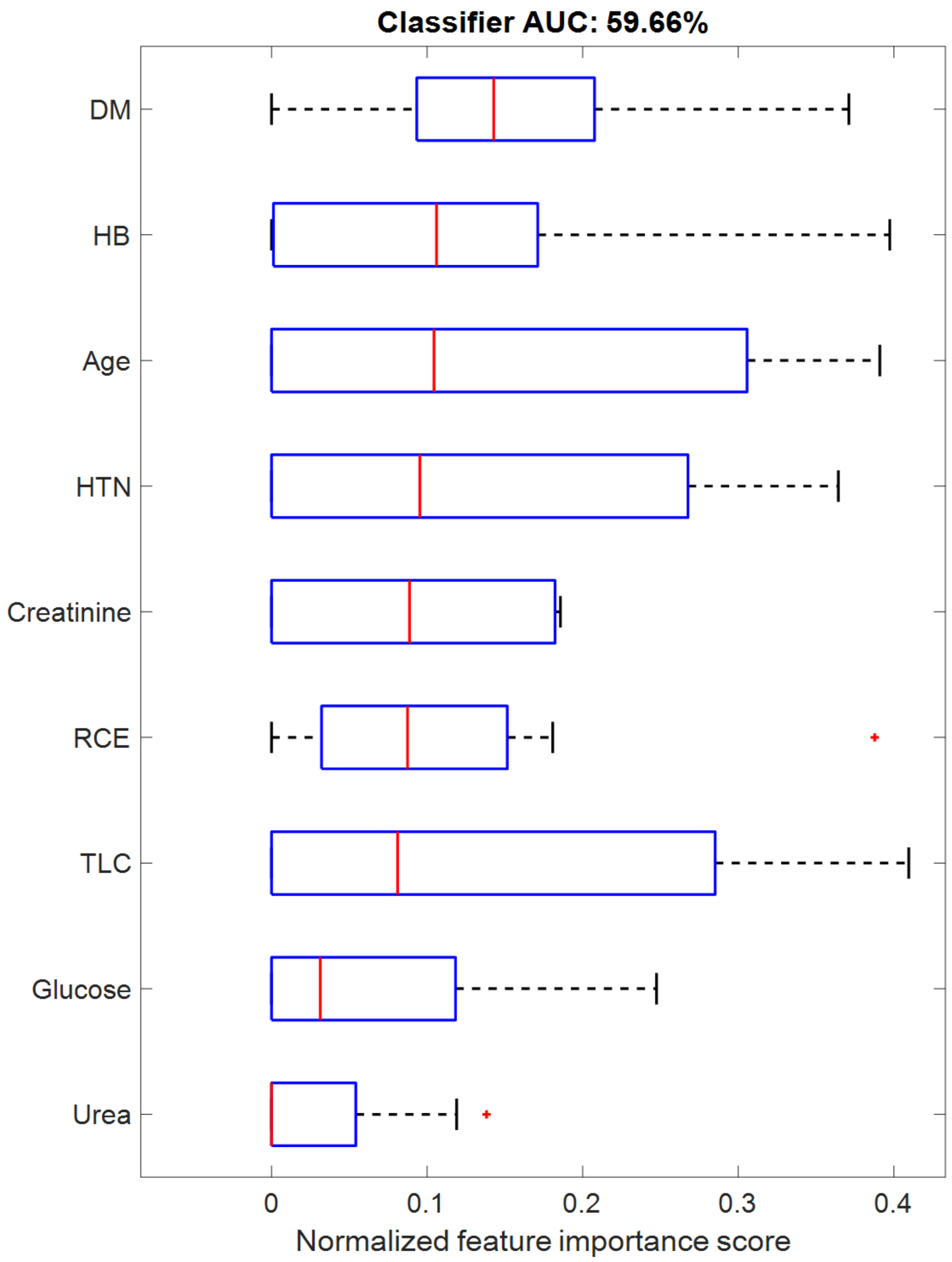

Feature importance of pulmonary embolism classifier using feature set-6 (FS6) as input

Figure S4G

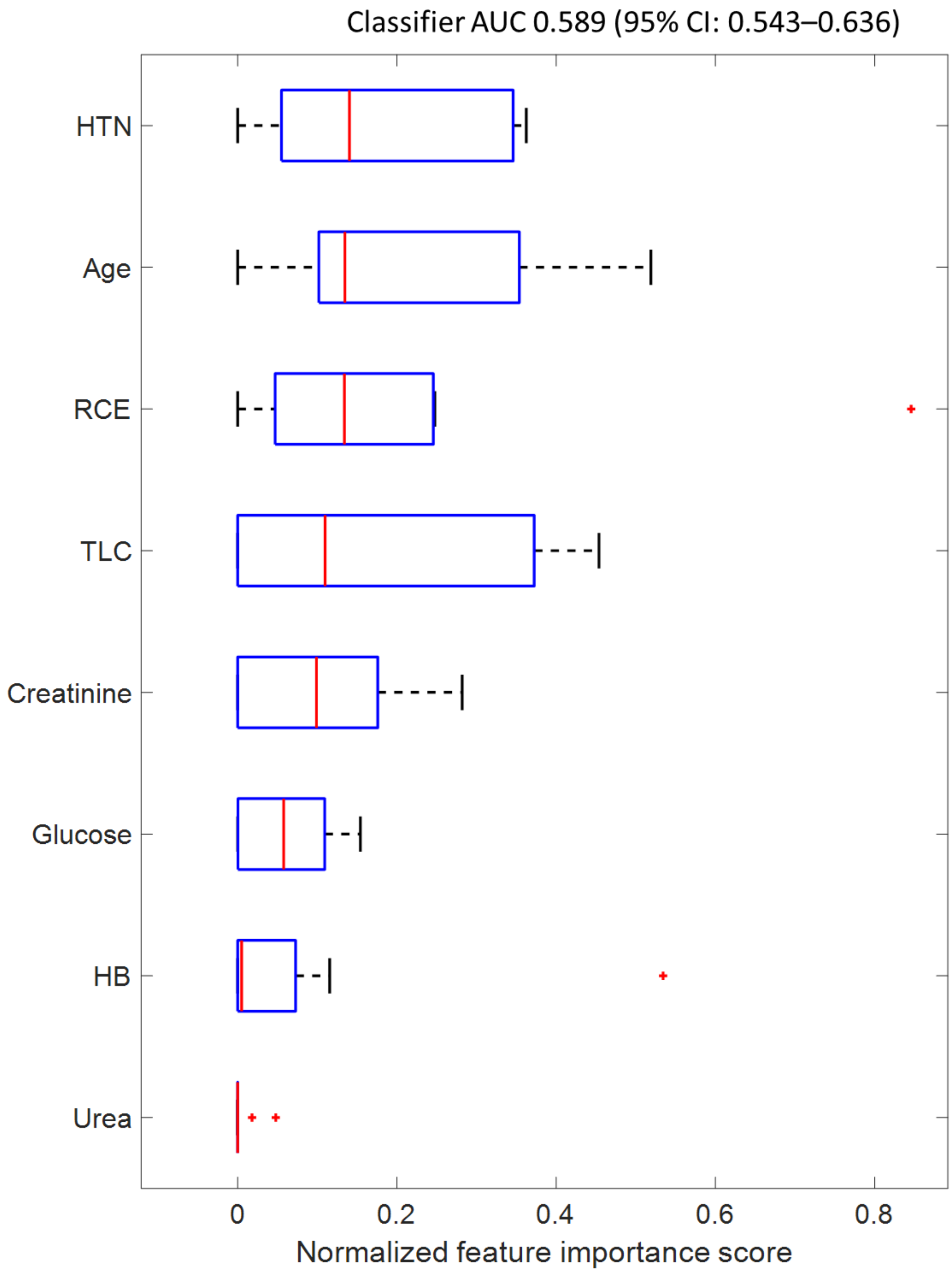

Feature importance of pulmonary embolism classifier using feature set-7 (FS7) as input

Online Supplement Figure S4. Feature importance scores for predicting of pulmonary embolism using (A) FS1; (B) FS2; (C) FS3; (D) FS4; (E) FS5; (F) FS6; (G) FS7.

Figure S5A

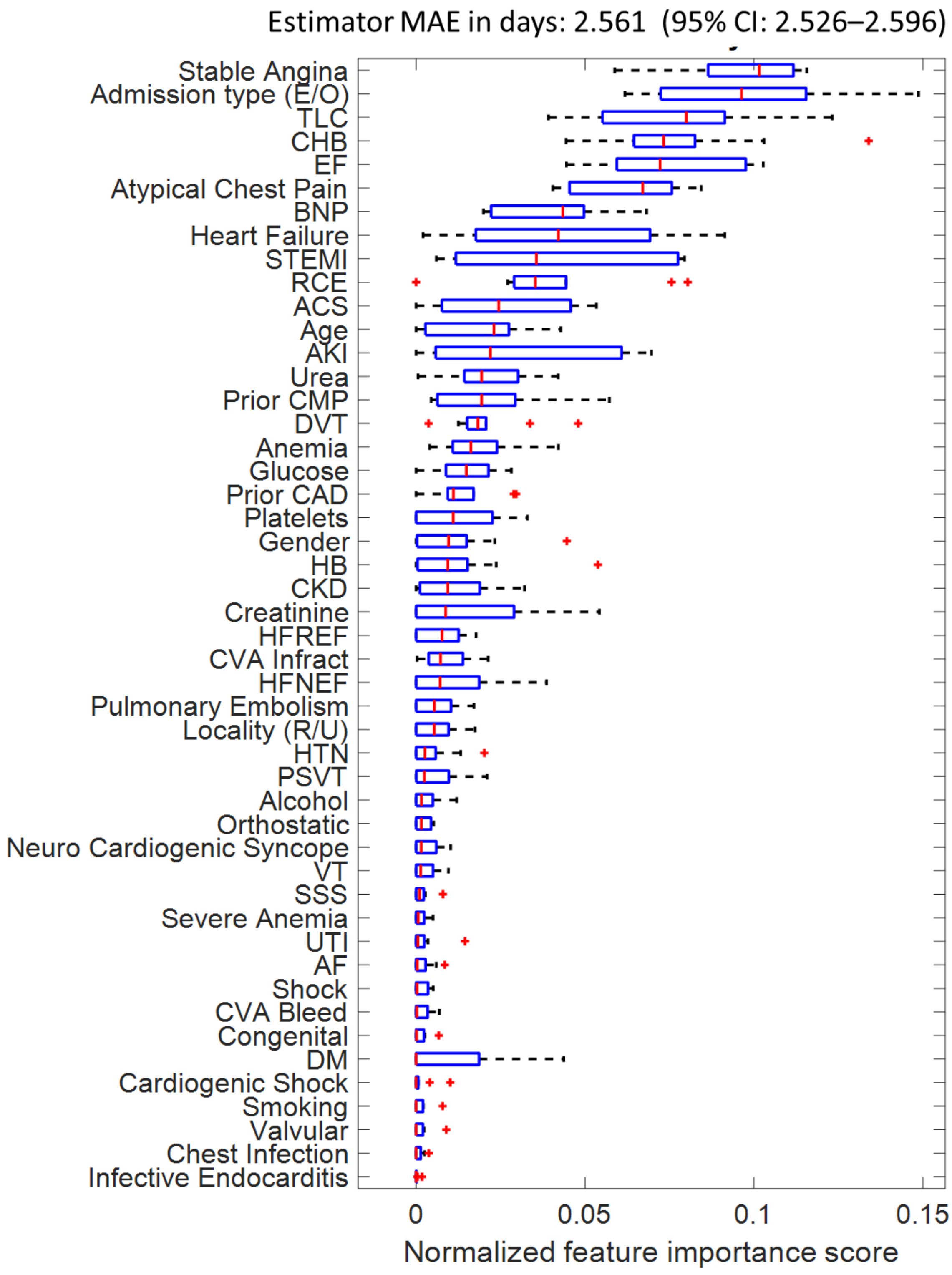

Feature importance of duration of stay estimator using feature set-1 (FS1) as input

Figure S5B

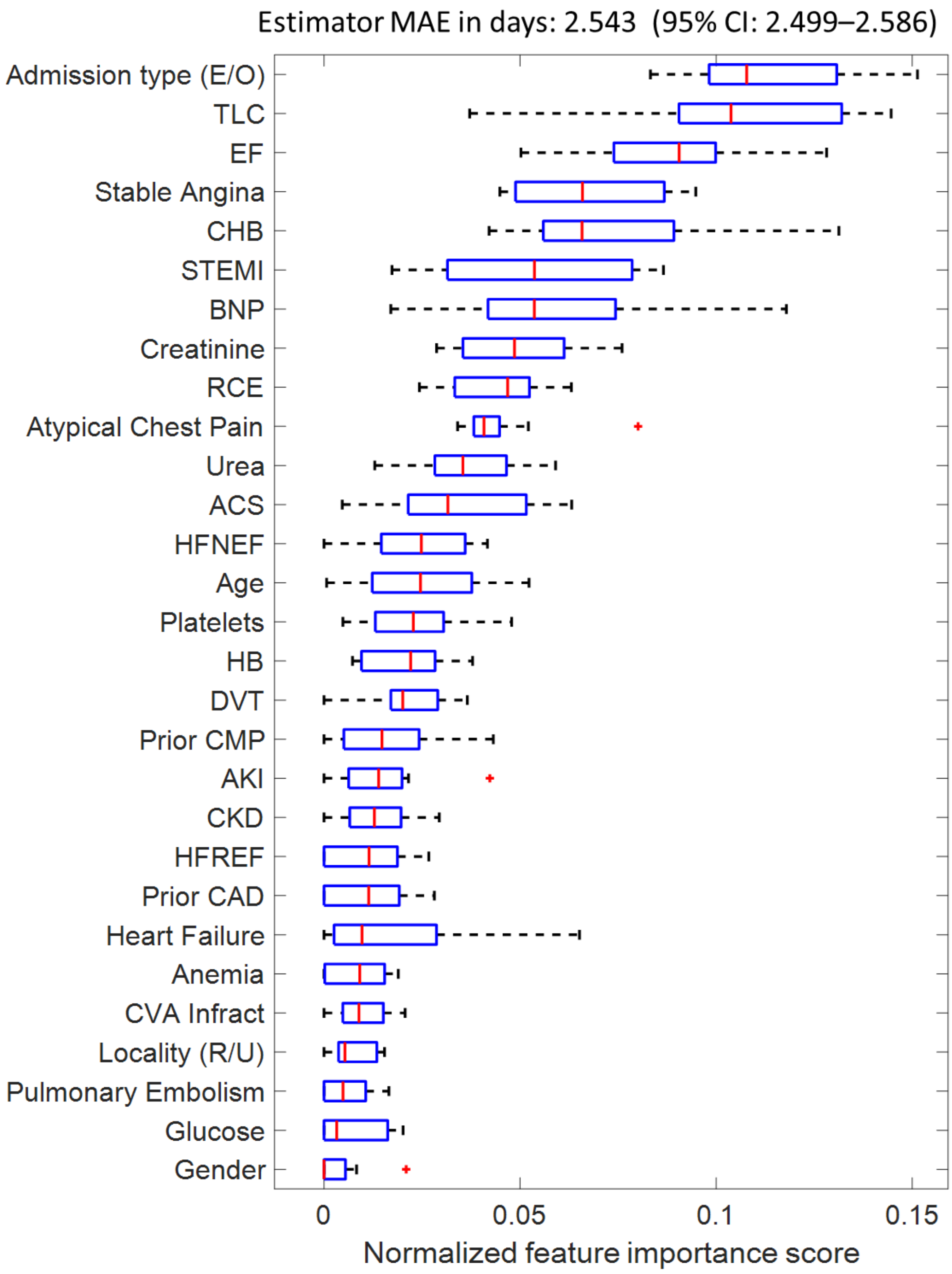

Feature importance of duration of stay estimator using feature set-2 (FS2) as input

Figure S5C

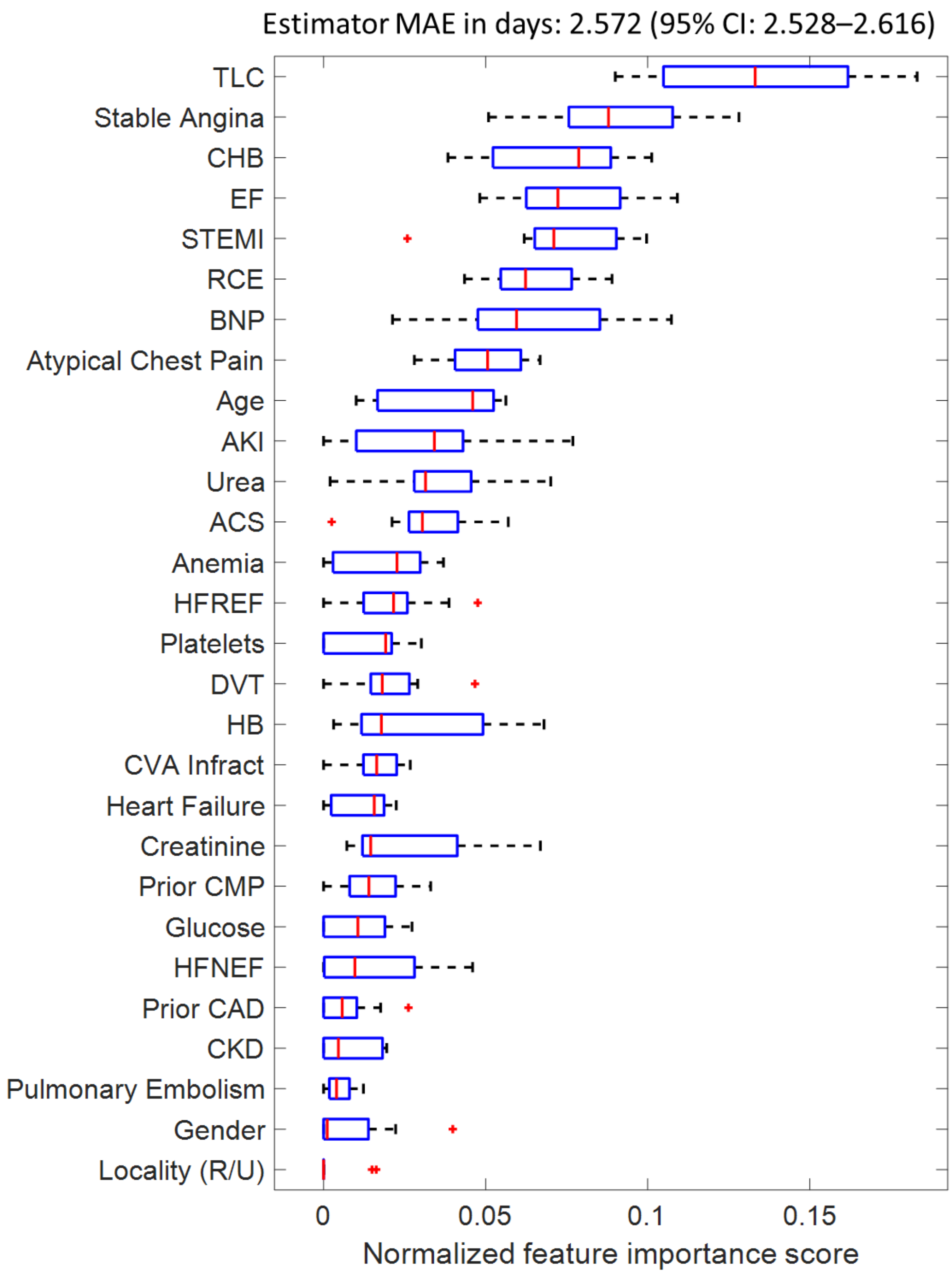

Feature importance of duration of stay estimator using feature set-3 (FS3) as input

Figure S5D

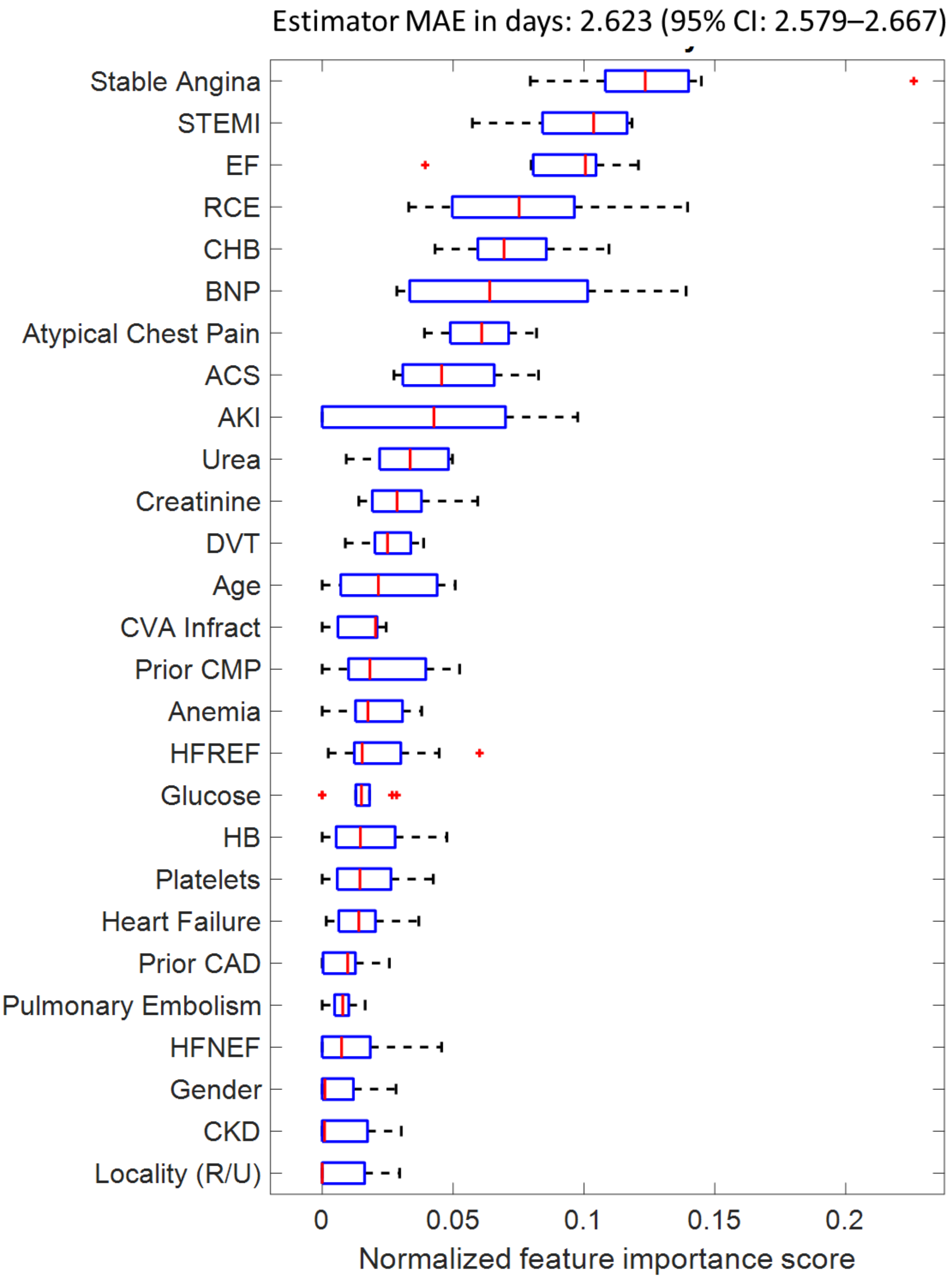

Feature importance of duration of stay estimator using feature set-4 (FS4) as input

Figure S5E

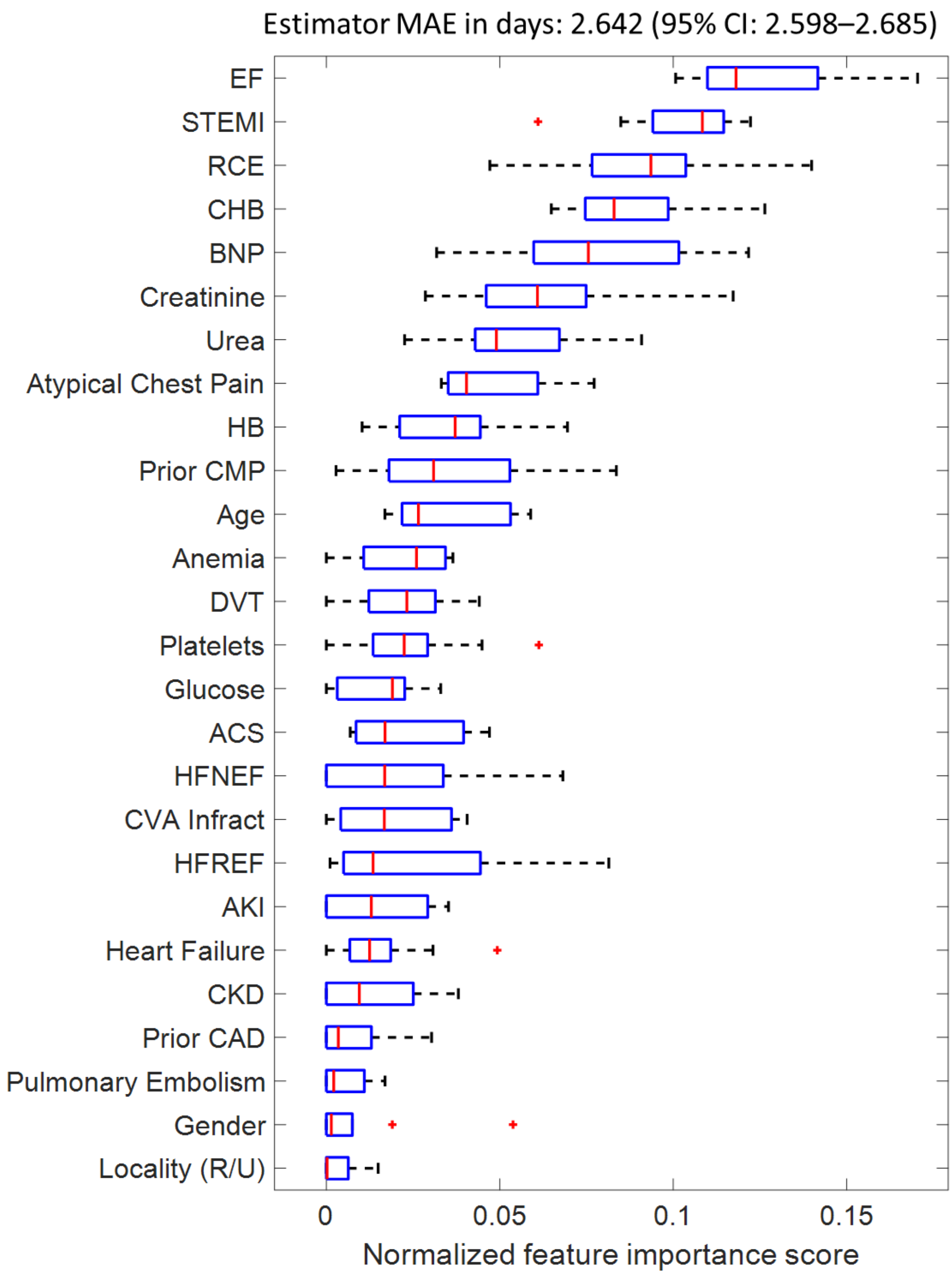

Feature importance of duration of stay estimator using feature set-5 (FS5) as input

Figure S5F

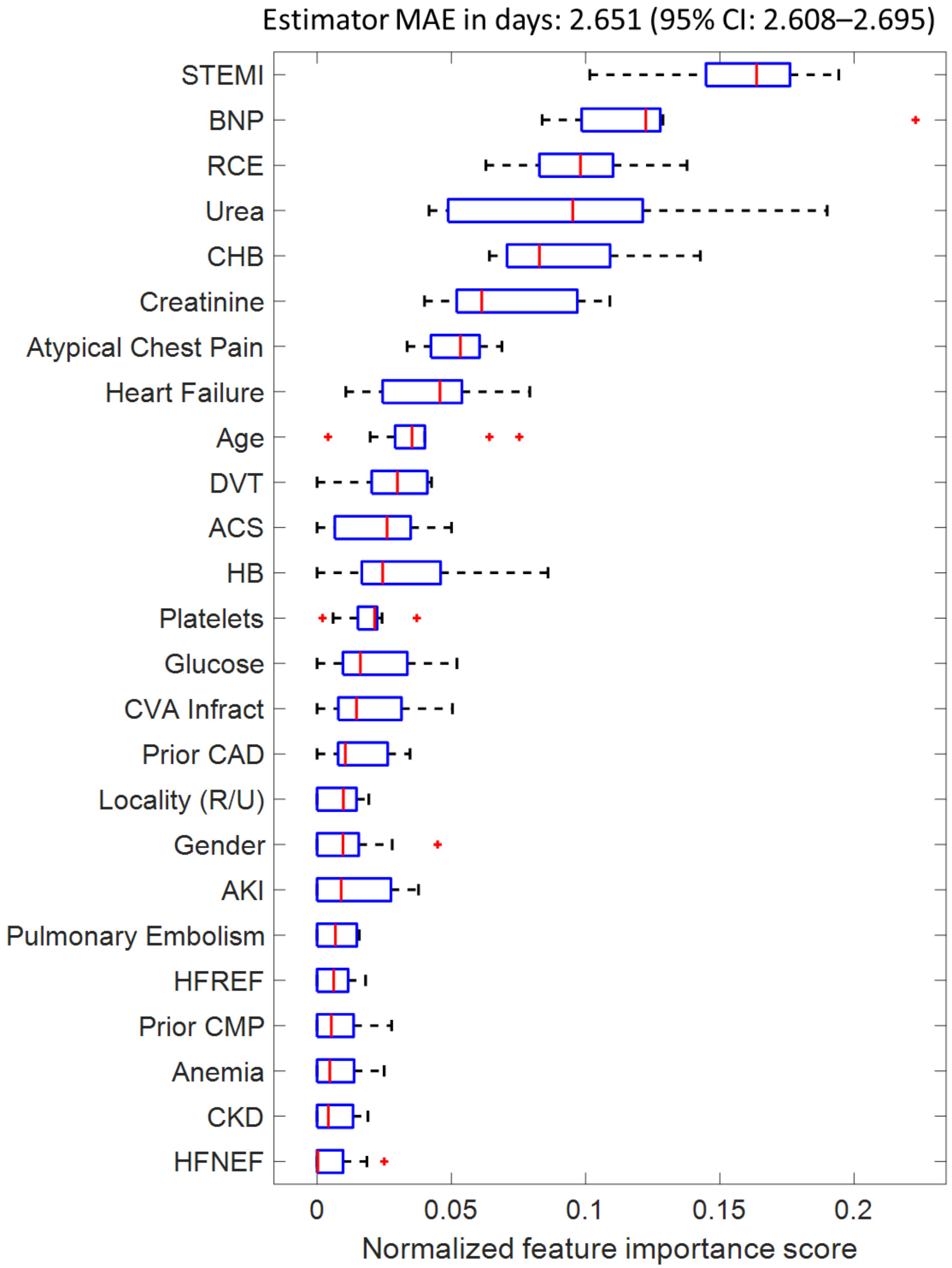

Feature importance of duration of stay estimator using feature set-6 (FS6) as input

Figure S5G

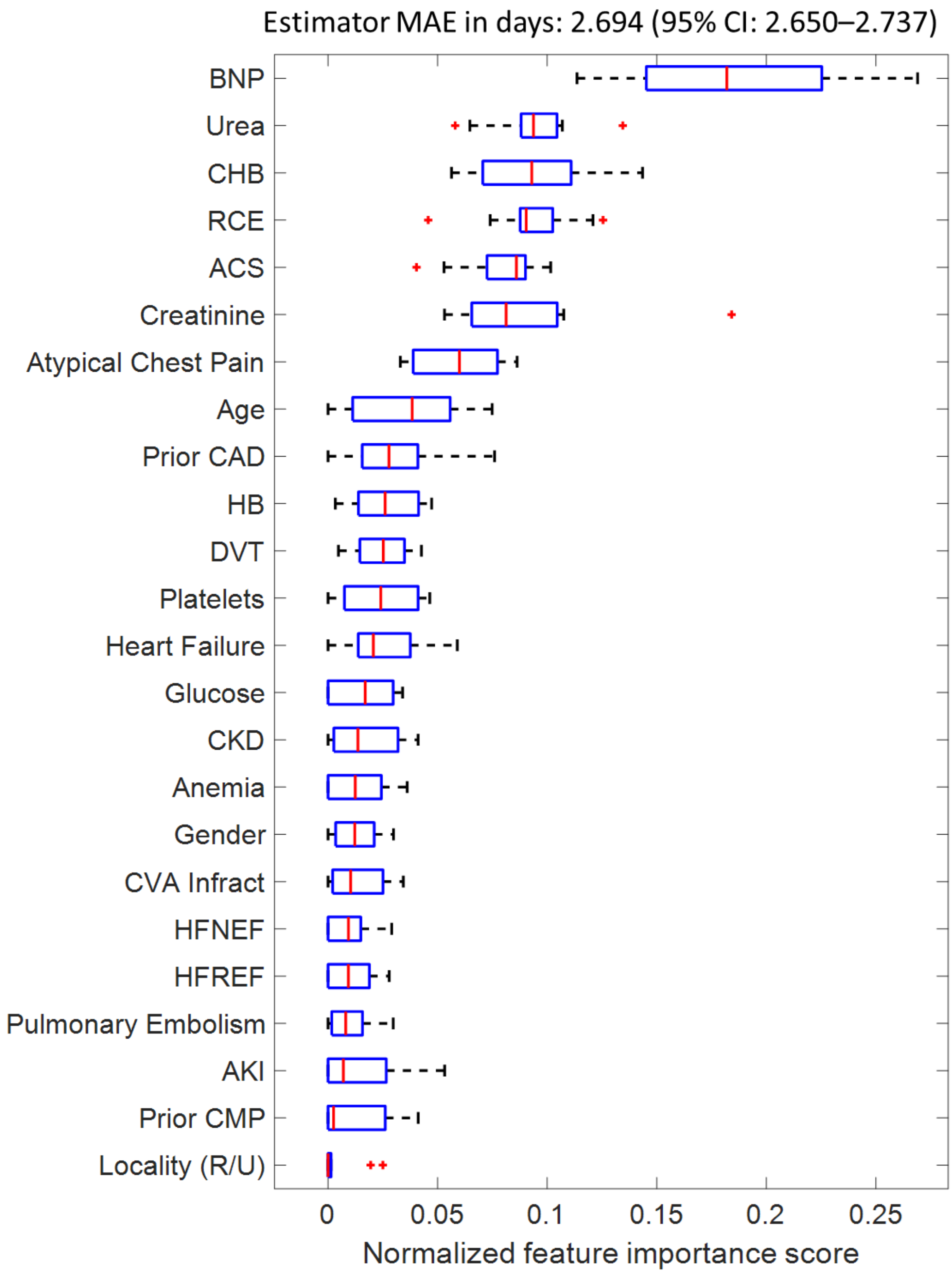

Feature importance of duration of stay estimator using feature set-7 (FS7) as input

Online Supplement Figure S5. Feature importance scores for estimating duration of stay using (A) FS1; (B) FS2; (C) FS3; (D) FS4; (E) FS5; (F) FS6; (G) FS7.

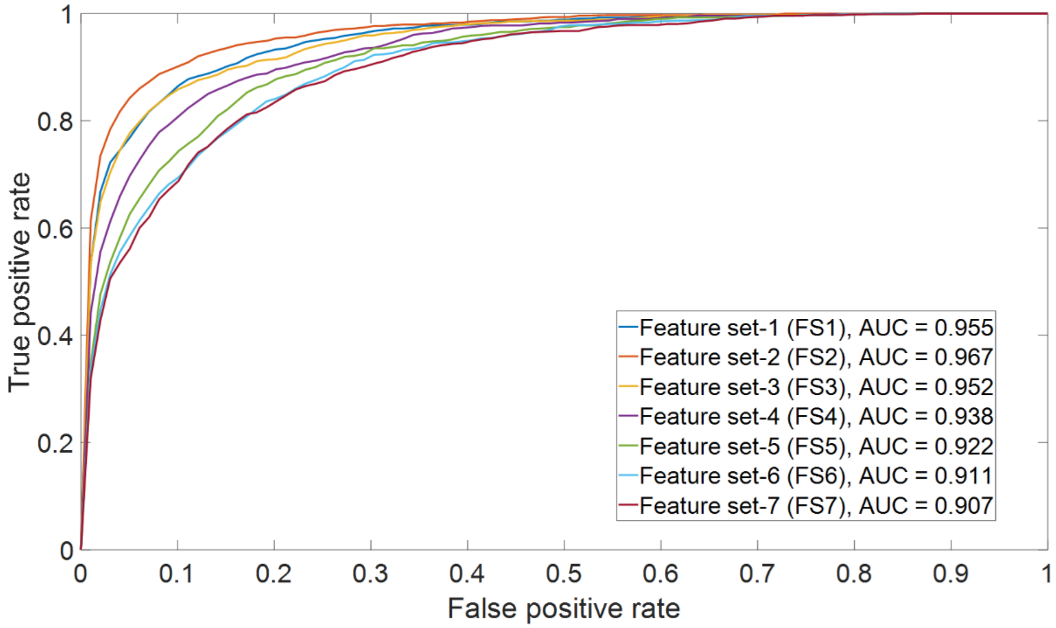

Online Supplement Figure S6. Comparison of receiver operation characteristic (ROC) curves of mortality classifier using feature sets FS1-FS7 as inputs. The classifier model using FS2 as input has superior performance over the model using FS1 as input, and the performance gradually decreases with input being varied from FS3 to FS7.

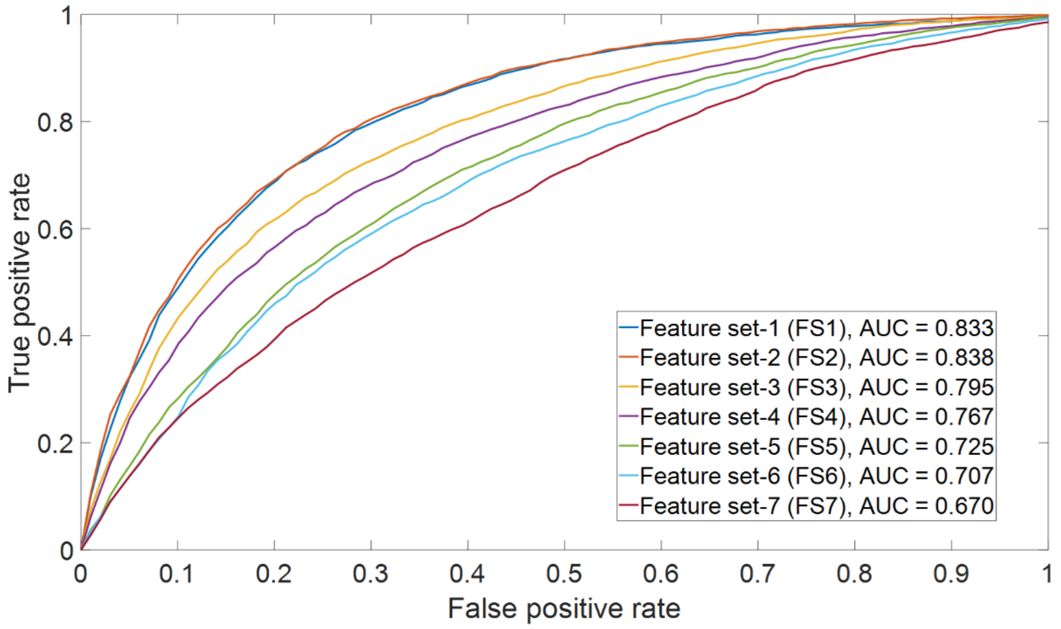

Online Supplement Figure S7. Comparison of ROC curves of heart failure classifier using feature sets FS1-FS7 as inputs. The classifier model using FS2 as input has slightly better performance over the model using FS1 as input, and the performance gradually decreases with input being varied from FS3 to FS7.

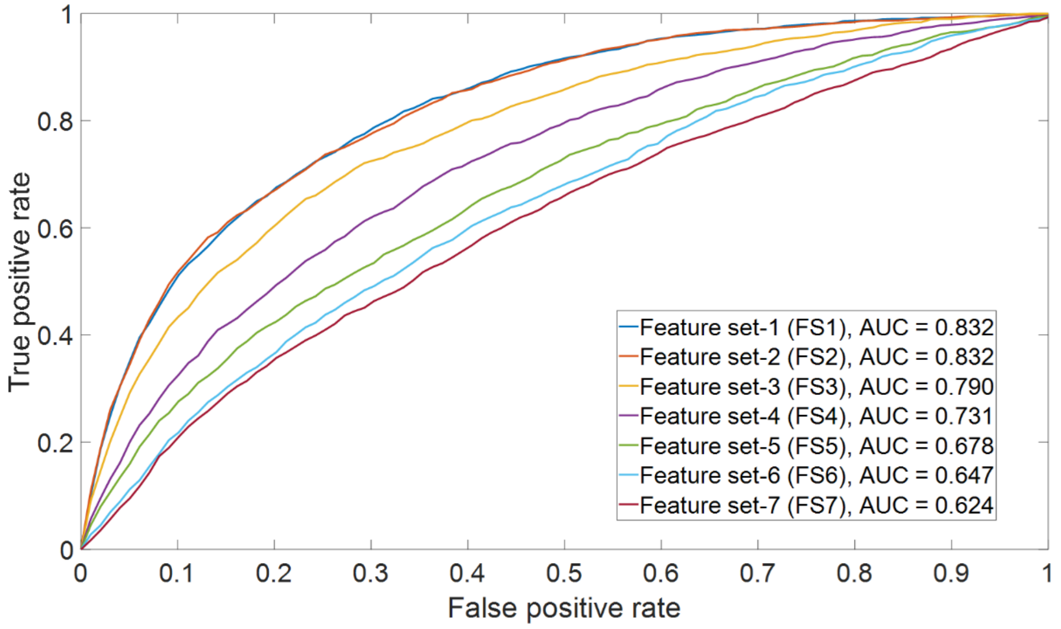

Online Supplement Figure S8. Comparison of ROC curves of ST-segment elevation myocardial

infarction (STEMI) classifier using feature sets FS1-FS7 as inputs. The classifier model using FS2 as input is comparable to the model using FS1 as input, and the performance gradually decreases with input being varied from FS3 to FS7.

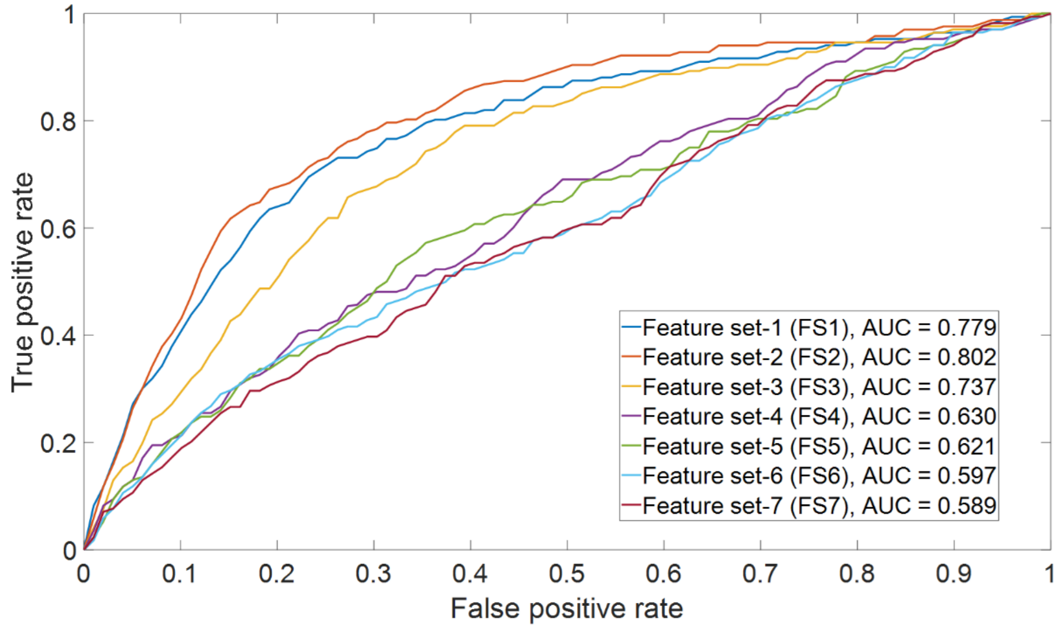

Online Supplement Figure S9. Comparison of ROC curves of pulmonary embolism classifier using feature sets FS1-FS7 as inputs. The classifier model using FS2 as input has superior performance over the model using FS1 as input, and the performance gradually decreases with input being varied from FS3 to FS7.
